# Evolution of fatty acid taste in drosophilids

**DOI:** 10.1016/j.celrep.2023.113297

**Published:** 2023-10-31

**Authors:** Manali Dey, Elizabeth Brown, Sandhya Charlu, Alex Keene, Anupama Dahanukar

**Affiliations:** 1 Interdepartmental Neuroscience Program, University of California, Riverside, Riverside, CA 92521, USA; 2 Department of Biology, Texas A&M University, College Station, TX 77843, USA; 3 Biomedical Sciences Graduate Program, University of California, Riverside, Riverside, CA 92521, USA; 4 Department of Molecular, Cell & Systems Biology, University of California, Riverside, Riverside, CA 92521, USA; 5 Lead contact

## Abstract

Comparative studies of related but ecologically distinct species can reveal how the nervous system evolves to drive behaviors that are particularly suited to certain environments. *Drosophila melanogaster* is a generalist that feeds and oviposits on most overripe fruits. A sibling species, *D. sechellia*, is an obligate specialist of *Morinda citrifolia* (noni) fruit, which is rich in fatty acids (FAs). To understand evolution of noni taste preference, we characterized behavioral and cellular responses to noni-associated FAs in three related drosophilids. We find that mixtures of sugar and noni FAs evoke strong aversion in the generalist species but not in *D. sechellia*. Surveys of taste sensory responses reveal noni FA- and species-specific differences in at least two mechanisms—bitter neuron activation and sweet neuron inhibition–that correlate with shifts in noni preference. Chemoreceptor mutant analysis in *D. melanogaster* predicts that multiple genetic changes account for evolution of gustatory preference in *D. sechellia*.

## In brief

Dey et al. characterize evolution of noni acid taste in drosophilids, identifying fatty acid- and species-specific differences in sweet- and bitter-sensing circuits that correlate with differences in feeding preference for noni acids between noni host-specialist and generalist species.

## Graphical abstract

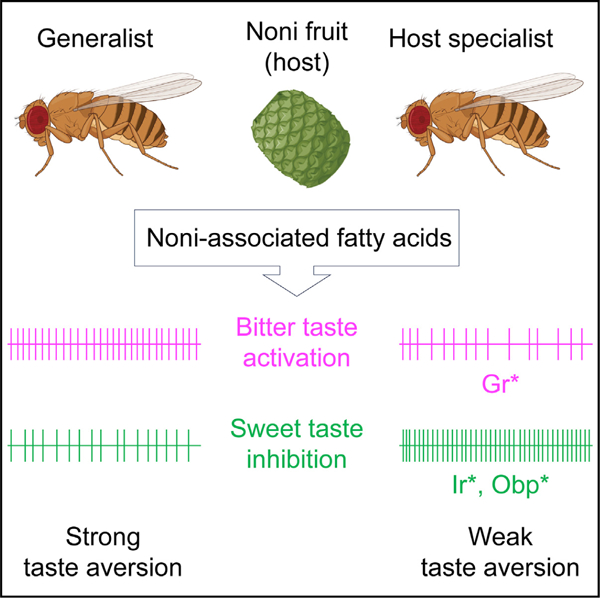

## INTRODUCTION

Dietary preferences differ dramatically between insect species, even among those that might be closely related, and they have large consequences on adaptive evolution. Host plants present a wealth of chemical information, and previous studies have found examples of both olfactory and gustatory variation that accompany adaptive responses to ecological niches.^1–9^ However, a central and poorly understood question is how chemosensory evolution contributes to differences in dietary preference. Insects that are host plant specialists present unique opportunities to decipher how the nervous system evolves to affect behavior.

*D. sechellia* is one such model, because of its obligate host specialization as well as its evolutionary proximity to *D. melanogaster*.^8^ *Drosophila* species originated in equatorial Africa from where they spread all over the world.^10^ The melanogaster subgroup contains nine sibling species. While most are generalists and can thrive on a broad range of hosts, two species, *D. sechellia* and *D. erecta*, depend on selected hosts, *Morinda citrifolia* (noni) and *Pandanus candelabrum*, respectively, for their survival.^5,11,12^ *D. sechellia* is endemic to the Seychelles archipelago in the Indian Ocean, where it exclusively feeds and oviposits on the fruit of *M. citrifolia* of the Rubiaceae family.^13,14^ An ancestor of *D. sechellia* is thought to have arrived on the Seychelles from the coast of Africa or Madagascar, and it diverged into *D. simulans, D. sechellia*, and *D. mauritiana.* Though *D. sechellia* diverged from *D. simulans* several thousand years ago,^15,16^ it is possible that its specialization for noni fruit may be a more recent development.^10,11^ The robust differences in dietary preference between *D. sechellia* and closely related drosophilid species allow for comparative approaches to define the neural mechanisms underlying preference for noni fruit.

Noni fruit is toxic for all other drosophilids found on the Seychelles, which is thought to provide an additional advantage to the specialist.^11,12,17–19^ The toxicity is largely attributed to medium chain fatty acids (FAs), which along with additional carboxylic acids and derivatives comprise a significant fraction of the volatiles emanating from ripe noni fruit.^19^ Octanoic acid (OA) and hexanoic acid (HA) are major components (58% and 19% of the volatiles, respectively) and are individually toxic to the generalist species.^19^ Decanoic acid (DA), found in smaller amounts (1.54% of the volatiles), does not by itself have an observable effect on survival. However, a mixture of the three acids in the same proportions as present in noni causes the same mortality as fruit pulp, an effect that cannot be replicated with a mixture of OA and HA only.^17,19^ Thus, although the molecular basis of physiological tolerance to noni components is not yet well understood, it appears that *D. sechellia* has evolved detoxification mechanisms to cope with multiple chemical components of noni.^11,20–24^ The primary locus responsible for this resistance has been mapped to a cluster of 18 genes, including various *Obps* (odorant-binding proteins) and nine members of the *Osiris* (*Osi*) family.^20,25^ *Osi* genes encode conserved transmembrane proteins that are involved in various functions such as vesicular protein trafficking and degradation, olfactory cuticular nanopore formation, and pheromone sensitivity.^26–30^ *RNAi* knockdown of *Osi6*, *Osi7*, and *Osi8* in adult *D. melanogaster* alters its resistance to OA.^23^ Knockdown of *Osi8* also decreases OA resistance in larvae.^31^ Further investigation is needed to determine the mechanisms of how Osi proteins contribute to noni FA resistance.

While physiological tolerance is key for survival on noni fruit, accompanying changes in behavioral preference are important for driving interactions between *D. sechellia* and noni. *D. sechellia* is attracted to noni fruit, or mixtures of FAs and esters that mimic noni, from as far away as 150 m.^12,32^ Contact-range behaviors are also markedly different—*D. melanogaster* and *D. simulans* avoid feeding and laying eggs on substrates containing noni FAs, whereas *D. sechellia* females choose to do so.^33–36^ Several studies have investigated mechanisms underlying olfactory adaptations that increase attraction of *D. sechellia* to noni volatiles and also induce oviposition.^12,32,37,38^ However, little is known about neurogenetic variation in gustatory function, which is engaged upon contact with the fruit.

Investigations of FA taste in *D. melanogaster* have largely focused on appetitive responses elicited by HA,^39^ which is mediated via a subset of sweet-sensing taste neurons and is dependent on members of the ionotropic receptor family, including co-receptors Ir25a and Ir76b, as well as a selectively expressed Ir56d.^40–42^ Whether these functions are altered in *D. sechellia* has not been explored. Comparative studies have delved more into gustatory-driven oviposition behavior and found FA aversion in the generalists, contrasted with preference in *D. sechellia*.^12,18,43^ Loss of *Obp57d* and *Obp57e* or knockdown of *Obp56e* in *D. melanogaster* causes reduced aversion to noni fruit.^33–36^ Obps are widely expressed in taste tissues and are involved in modulating feeding behaviors to various stimuli.^44–47^ Yet, how these Obps act to alter FA taste responses is not yet clear. Overall, these questions reflect a significant gap in our understanding, since changes in taste and dietary preference may have been key events that facilitated selection of increased behavioral attraction and physiological adaptations in *D. sechellia*.^11,48^

Here, we systematically characterize gustatory responses of *D. sechellia* and its generalist siblings, *D. simulans* and *D. melanogaster*. We find major evolutionary shifts in behavioral responses to the three noni FAs. We test labellar sensilla for responses to noni FAs, and we find that noni FAs activate bitter taste neurons and also inhibit sweet taste neurons. Further, we find noni FA- and species-specific differences in these features that parallel evolutionary shifts in feeding preference. Analysis of *D. melanogaster* chemoreceptor mutants suggests that a Gr-dependent mechanism is involved in bitter neuron activation, and an Ir-dependent mechanism is involved, at least in part, in sweet neuron inhibition. Overall, our study predicts that multiple variants impacting the function of at least two different classes of taste neurons allow *D. sechellia* to favor noni.

## RESULTS

### *D. sechellia* shows a loss of feeding aversion to noni FAs

**Figure 1.**
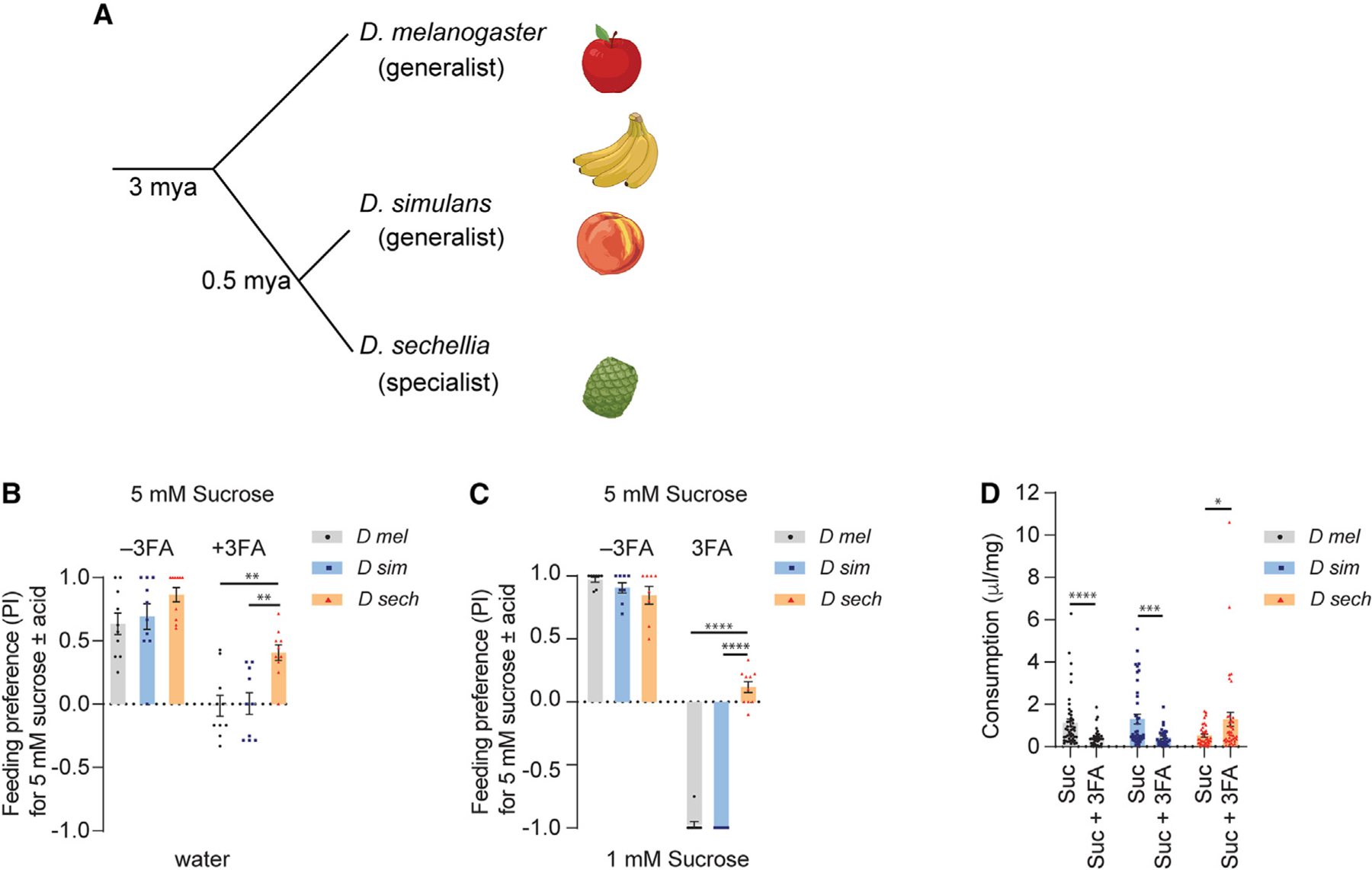
*D. sechellia* has higher taste preference for noni FAs compared to its generalist sister species (A) Phylogenetic tree showing the evolutionary relationship between three *Drosophila* species. (B) Feeding preference of indicated *Drosophila* species to 5 mM sucrose with or without a mixture of three noni FAs (3FA; 1% octanoic acid, 0.5% hexanoic acid, 0.05% decanoic acid) tested against water. n = 10. (C) Feeding preference of indicated *Drosophila* species to 5 mM sucrose with or without 3FA (as in B) tested against 1 mM sucrose. n = 8–10. (D) Ingested volume (μL) of indicated diet normalized to body weight (mg) in mated females. *D mel*, n = 48 (5 mM sucrose), n = 39 (5 mM sucrose + 3FA); *D sim*,n= 42 (5 mM sucrose), n = 30 (5 mM sucrose + 3FA); *D sech*, n = 36 (5 mM sucrose), n = 38 (5 mM sucrose + 3FA). Data were analyzed using two-way ANOVA with Tukey’s post hoc multiple comparisons test (B and C) and t test with Welch’s correction (D), *p < 0.05, **p < 0.01, ***p < 0.001, ****p < 0.0001. Error bars represent SEM. See also Figure S1.

We examined gustatory behaviors of three *Drosophila* species separated from each other by less than 3 million years—*D. melanogaster*, *D. simulans*, and *D. sechellia* (Figure 1A)—to FAs found in noni fruit. Since previous studies have shown that HA triggers proboscis extension response (PER) in *D. melanogaster*,^39,41,49^ we compared PER to noni FAs. We tested the three noni FAs separately, each at two different concentrations, the higher of which approximates that found in noni fruit.^19^ Consistent with previous studies, *D. melanogaster* showed proboscis extension in response to tarsal stimulation with noni FAs (Figures S1A–S1C). As expected, this appetitive PER, as well as consumption, in response to pure HA was dependent on *Ir56d* (Figures S1D and S1E). But we found little to no difference in PER to OA or HA across the three species, although *D. sechellia* exhibited a somewhat stronger PER to DA than the generalists.

Since FAs are found in the presence of sugar in noni fruit, we next performed binary choice feeding assays in which one tastant alternative was 5 mM sucrose mixed with OA, HA, and DA at concentrations that mimic the approximate levels present in ripe noni fruit (1% OA, 0.5% HA, and 0.05% DA).^19^ The sucrose-FA mixture was tested with water as the alternative. *D. sechellia* exhibited a much greater preference for sucrose-FA compared to either of the two generalist species (Figure 1B). We next tested gustatory preference for the sucrose-FA mixture when the alternative was 1 mM sucrose. The lower concentration of sucrose is less appetitive than 5 mM sucrose, but it can be preferred when aversive tastants are mixed with 5 mM sucrose. In these experiments, we found that *D. sechellia* did not distinguish between the two stimuli; however the generalists showed complete preference for 1 mM sucrose, indicating that noni FAs reduce food palatability for *D. melanogaster* and *D. simulans* (Figure 1C). Consistent with these results, we found that consumption of food containing noni FAs was significantly reduced in the generalist species (Figure 1D). By contrast, the presence of noni FAs stimulated food intake in *D. sechellia*. Overall, our results suggest that sucrose-noni FA mixtures elicit behavioral aversion in the generalist species, which is greatly reduced in *D. sechellia*.

### Individual noni FAs elicit species-specific feeding preference

**Figure 2.**
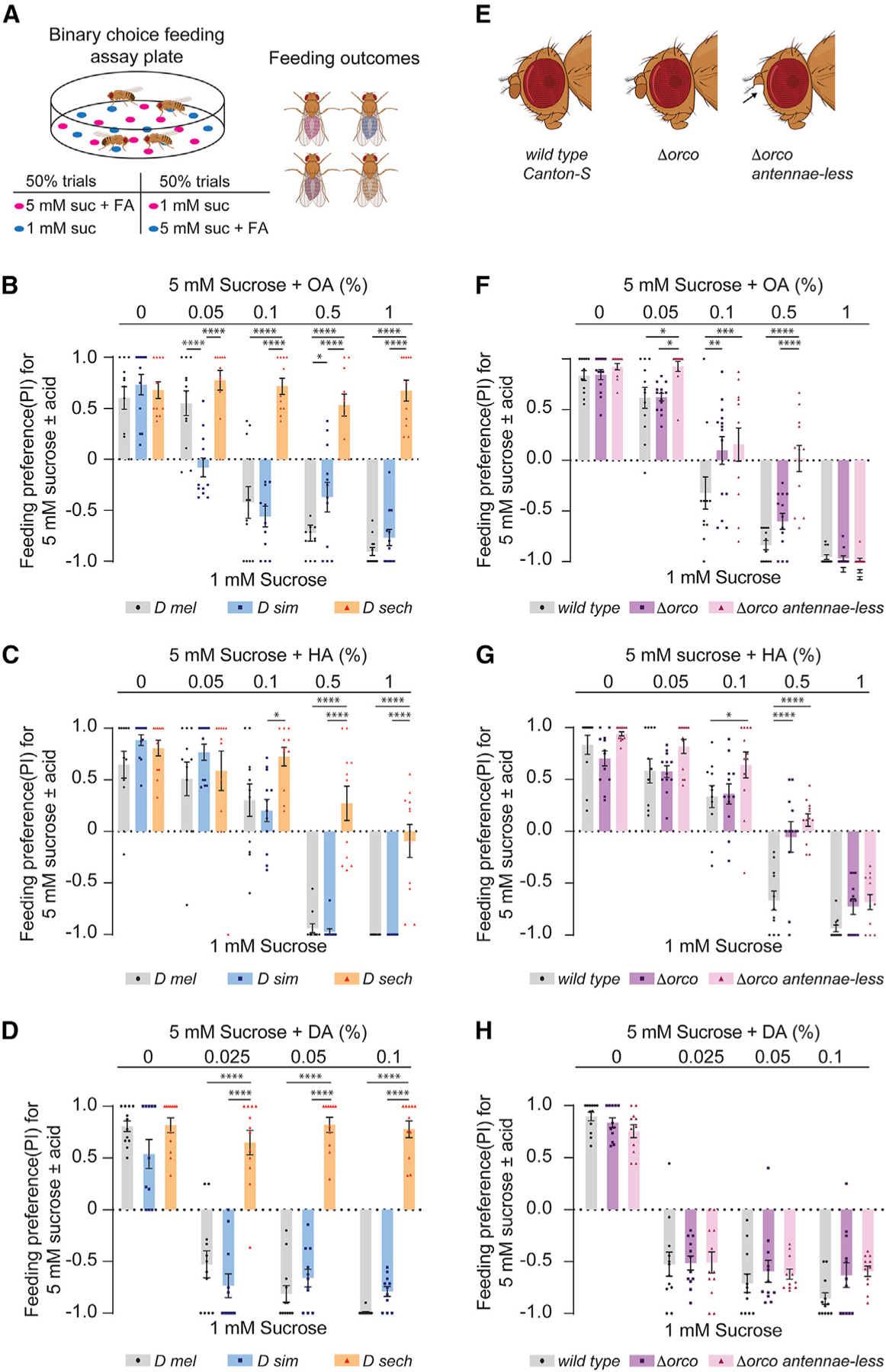
Noni FAs evoke species-specific feeding preferences (A) Diagram of binary feeding assay used for (B)–(D) and (F)–(H) outcomes. Created with BioRender.com. (B–D) Feeding preference of indicated species for mixtures of 5 mM sucrose with varying concentrations of octanoic acid (OA), hexanoic acid (HA), or decanoic acid (DA) tested against 1 mM sucrose. n = 12 trials for each concentration of the indicated FA. (E) Flies used for results shown in (F)–(H): Canton-S (wild-type), *orco*^1^ (∆*orco*), or *orco*^1^ with the third segment of the antennae removed surgically (∆*orco* antennae-less). Created with BioRender.com. (F–H) Feeding preference of indicated flies for 5 mM sucrose with varying concentrations of OA (F, n = 12–14), HA (G, n = 11–12), or DA (H, n = 11–12) tested against 1 mM sucrose. Data were analyzed using two-way ANOVA with Tukey’s post hoc multiple comparisons test, *p < 0.05, **p < 0.01, ***p < 0.001, ****p < 0.0001. Error bars represent SEM.

It is possible that all three noni FAs contribute to differences in the response of *D. sechellia*, or that a single FA drives the change in preference. Since the differences between generalist and specialist species in terms of their feeding preference for the noni FA mixture was more pronounced in binary choice assays with sucrose, we compared behavioral responses to individual noni acids under the same conditions (Figure 2A). Varying concentrations of each of the three acids were mixed with 5 mM sucrose and tested against 1 mM sucrose. FA concentrations were selected based on the range reported for noni fruit^17,19^ and, in some cases, previous reports of behavioral sensitivity.^18,33,34,36^ Overall, we found species-specific patterns of feeding preference for individual noni FAs. For *D. melanogaster* and *D. simulans*, all three FAs caused concentration-dependent shifts in feeding preference away from the sucrose-FA mixtures and toward the lower concentration of plain sucrose instead (Figures 2B–2D). Preference was also determined by FA identity, with DA being the least preferred of the three (Figure 2D). Interestingly, the behavioral threshold for OA was lower in *D. simulans*, suggesting enhanced sensitivity compared to that of *D. melanogaster* (Figure 2B). By contrast, inclusion of neither OA nor DA influenced preference of *D. sechellia* for the higher concentration of sucrose. Some reduction in preference was observed with higher concentrations of HA, but nevertheless, *D. sechellia* always preferred the sucrose-HA mixtures to a greater extent than the sister species (Figure 2C). Overall, these results indicate that the generalist drosophilids have far less preference for the three prominent FAs in noni fruit compared to *D. sechellia*.

### Anosmic *D. melanogaster* retain behavioral avoidance of noni FAs

The noni FAs are volatile, and there are differences in olfactory responses to HA and OA between *D. melanogaster* and *D. sechellia*, attributable to differences in olfactory receptors such as Or22a and Ir75b, as well as in olfactory circuits.^12,32,37,38^ Although olfaction-independent FA taste responses have been described, these have largely been investigated in the context of appetitive behavior.^39–42,49,50^ To determine if olfactory input contributes to FA preferences, we compared the behavior of wild-type *D. melanogaster* with that of *Orco* mutants (∆*Orco*) lacking *Or*-dependent olfactory sensing, and with antennaeless *Orco* mutants (∆*Orco* antennae-less) lacking all olfactory input^51^ (Figure 2E). Flies were tested for feeding preference for sucrose-noni FA mixtures using the binary choice assay conditions described above. We found that all three FAs elicited reduced preference at the highest concentrations, even in flies that had lost the ability to smell them (Figures 2F–2H). In fact, DA aversion was not significantly different between the control and experimental groups at any of the tested concentrations (Figure 2H); thus, DA feeding avoidance, at least across the tested concentrations, is independent of olfactory function. For OA and HA, on the other hand, olfaction-impaired flies showed reduced avoidance of the sugar-FA mixtures, indicating a partial contribution of olfactory input to the behavioral outcomes in this assay (Figures 2F and 2G). Therefore, in generalist species, both olfactory and taste functions contribute to the feeding avoidance of noni FAs.

### Sensillar responses to noni FAs differ between species

**Figure 3.**
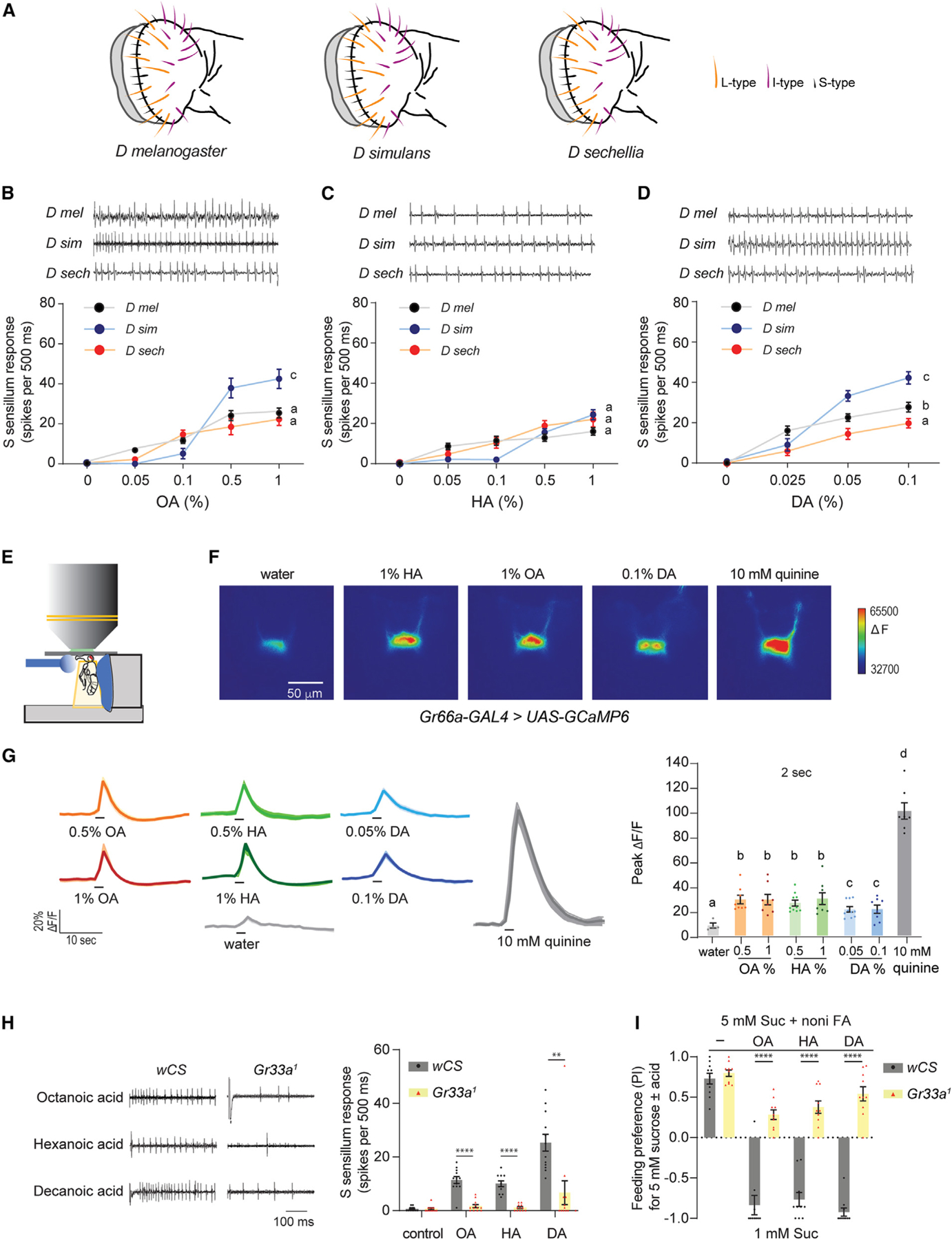
Noni FAs activate bitter neurons in S-type sensilla (A) Arrangement of sensillar types in the labella of three *Drosophila* species. (B–D) Representative traces and mean responses of labellar S-type sensilla in indicated species to varying concentrations of octanoic acid (B, n = 10–14), hexanoic acid (C, n = 9–15), and decanoic acid (D, n = 10–18). (E) Diagram of set up for live imaging of tastant-evoked changes in GCaMP6 fluorescence in axon terminals. Stimuli are applied to the proboscis. (F) Representative pseudocolored images of calcium activity in axon terminals of *Gr66a*^+^ neurons in response to 2-s stimulation of the labellum with indicated tastant. Scale bar represents 50 μm. (G) Activity traces and average peak changes in GCaMP6 fluorescence in *Gr66a*^+^ neurons to 2-s applications of tastants. n = 4 (water), n = 8 (0.5% OA), n = 8 (1% OA), n = 10 (0.5% HA), n = 8 (1% HA), n = 10 (0.05% DA), n = 8 (0.1% DA), n = 7 (10 mM quinine). (H) Representative traces (left) and mean responses (right) obtained from S-type in *w Canton-S* (*wCS*) or *Gr33a*^1^ flies stimulated with the indicated tastants. Tastants were the following: octanoic acid (OA, 1%), hexanoic acid (HA, 1%), decanoic acid (DA, 0.1%), and tricholine citrate diluent (control, 30 mM). For control flies, n = 12 (control), n = 12 (OA), n = 11 (HA), and n = 12 (DA). For *Gr33a*^1^ flies, n = 12 (control), n = 12 (OA), n = 12 (HA), and n = 12 (DA). (I) Feeding preference of *D. melanogaster* flies to 5 mM sucrose mixed with octanoic acid (OC, 1%), hexanoic acid (HA, 1%), or decanoic acid (DA, 0.1%) tested against 1 mM sucrose. Genotypes are as in (H). n = 10. All representative traces show the first 500-ms period following contact between the stimulus and taste hair. Data in (B)–(D) were analyzed using two-way ANOVA for repeated measures with Tukey’s post hoc multiple comparisons test; data in (G) were analyzed with one-way ANOVA with Sidak’s post hoc test for multiple comparisons; data in (H) and (I) were analyzed with the unpaired t test with Welch’s correction. *p < 0.05, **p < 0.01, ***p < 0.001, ****p < 0.0001. Error bars represent SEM. See also Figures S2, S3, and S4.

To elucidate the sensory basis of gustatory preference in the generalist and specialist species, we surveyed responses to noni FAs in taste sensilla of the labellum. We focused on L- and S-type sensilla, which represent units that either contain or lack a bitter taste neuron within them. As reported previously,^52^ we found that both the numbers and positions of these sensilla were largely similar in the three species, facilitating comparative analyses (Figure 3A). From initial recordings, we found similar strengths of OA and HA responses between *D. melanogaster* and *D. sechellia* (Figures 3B–3D). However, DA responses were significantly lower in *D. sechellia* compared to *D. melanogaster* (Figure 3D) and correlated with reduced feeding aversion to DA (Figures 2D and 2H) and the 3FA mixture containing DA (Figures 1B and 1C). Interestingly, of the three species, *D. simulans* exhibited strongest responses to OA and DA, paralleling its heightened sensitivity to these compounds in binary choice feeding assays. Since S-type sensilla are also activated by bitter tastants, we compared responses to lobeline, denatonium, coumarin, and caffeine. We found that with the exception of caffeine, which, as previously reported, did not evoke a response in *D. sechellia*,^52^ responses to other bitter tastants were similar in the three species (Figure S3A).

### Noni FAs activate bitter taste neurons via gustatory receptors

The robust responses in Sa- and Sb-type sensilla mimic the pattern observed for ca- responses in Sa- and Sb-type sensilla; we therefore compared pooled S-type responses across species. For L sensilla, we primarily recorded from L7–9, which are positioned laterally to the S hairs. OA and HA were tested at a range of 0.05%–1% and DA from 0.025%–0.1%. We did not find robust dose-dependent spiking activity in response to any of the three noni FAs in the selected L-type sensilla of any species (Figure S2). On the other hand, spike trains consistent with a single responsive neuron were observed upon stimulation of S-type sensilla with noni FAs (Figures 3B–3D). In all three species, response to each FA exhibited dose dependence. We found no differences in nonical bitter tastants.^53^ We therefore hypothesized that the observed FA responses originate in bitter-sensing neurons, which are present in S-type but not in L-type sensilla.^53^ To test this idea, we measured 1% HA responses from S-type sensilla in flies in which either *Gr64f* sweet- or *Gr32a* bitter-sensing taste neurons were genetically silenced by expression of *Kir2.1*. As in wild-type flies, we observed a strong response to 1% HA in S-type sensilla of control *UAS-Kir2.1* flies (Figure S3B). We also measured responses to control tastants—100 mM sucrose (sweet), 10 mM lobeline (bitter), 10 mM denatonium (bitter), as well as TCC—which were as expected. We found that response to HA was maintained in *Gr64f*-silenced flies; the loss of response to sucrose but not to lobeline or denatonium confirmed the specific functional ablation of sweet-sensing neurons. Conversely, in *Gr32a*-silenced flies, we observed that responses to HA as well as to lobeline and denatonium were abolished, whereas that to sucrose was maintained (Figure S3B).

As an independent line of evidence for bitter taste activation by FAs, we expressed GCaMP6 in bitter neurons and imaged tastant-evoked changes in fluorescence in presynaptic terminals of these neurons in the sub-esophageal zone (Figures 3E–3G). As expected, calcium activity was elevated in these neurons when 10 mM quinine was applied to the labellum. Additionally, we observed calcium activity in response to 2-s applications of the noni FAs. Interestingly, a longer stimulation period of 10 s uncovered one peak of calcium activity following stimulus onset (ON response) and a second peak upon stimulus removal (OFF response) for quinine as well as the noni FAs (Figure S3C). Such responses have recently been described in response to acetic acid and lactic acid, in both sweet- and bitter-sensing taste neurons.^54,55^

Since a previous report attributed HA-evoked spiking activity in S-type sensilla to Gr64e function in sweet-sensing neurons,^49^ we tested a *Gr64a-f* mutant and confirmed that while sucrose response was lost, HA responsiveness was not affected in S-type sensilla (Figure S4A). In line with the electrophysiology results, *Gr64a-f* mutants failed to distinguish between two concentrations of sucrose in binary choice assays but exhibited feeding aversion to 1% HA (Figure S4B). We also tested mutants lacking *Ir56d*, which functions in sweet-sensing neurons. These mutants exhibited no differences in the robust HA-evoked firing activity observed in labellar S-type and I-type sensilla (Figures S4C–S4E). Moreover, *Ir56d* mutants, both intact and antennae-less, retained behavioral aversion to sucrose-HA mixtures in binary choice feeding experiments (Figures S4F and S4G).

We next tested *D. melanogaster* flies lacking the Gr33a receptor, which is expressed in all bitter taste neurons in the labellum and is required for responses to a number of bitter tastants.^52,53^ FA responses in S-type sensilla were nearly abolished in *Gr33a* mutants (Figure 3H). Concomitantly, *Gr33a* mutants exhibited much higher preference for sucrose-FA mixtures in binary choice assays, compared to control flies, which strongly avoided the mixtures (Figure 3I). Similar results were obtained when we tested an HA concentration range on *Gr33a* mutants in electrophysiology or behavior assays (Figures S4H and S4I). Collectively, our results, which are corroborated by a recent study,^56^ indicate that bitter taste neurons are strongly activated by the diverse FAs found in noni fruit.

### Noni FAs inhibit sweet-sensing neurons in a species-specific manner

Previous studies have found that some tastants activate bitter-sensing neurons and also inhibit sweet-sensing neurons, with both cellular mechanisms independently contributing to a fly’s avoidance of the tastant.^46,57–61^ We examined the responses of L-type sensilla, which did not exhibit any activity with noni FAs alone, to mixtures of sucrose with each of the noni FAs to test whether similar inhibitory mechanisms could be involved in determining behavioral outcomes to noni fruit. The noni FAs were tested across the same concentration range that was used to evaluate bitter taste activity.

**Figure 4.**
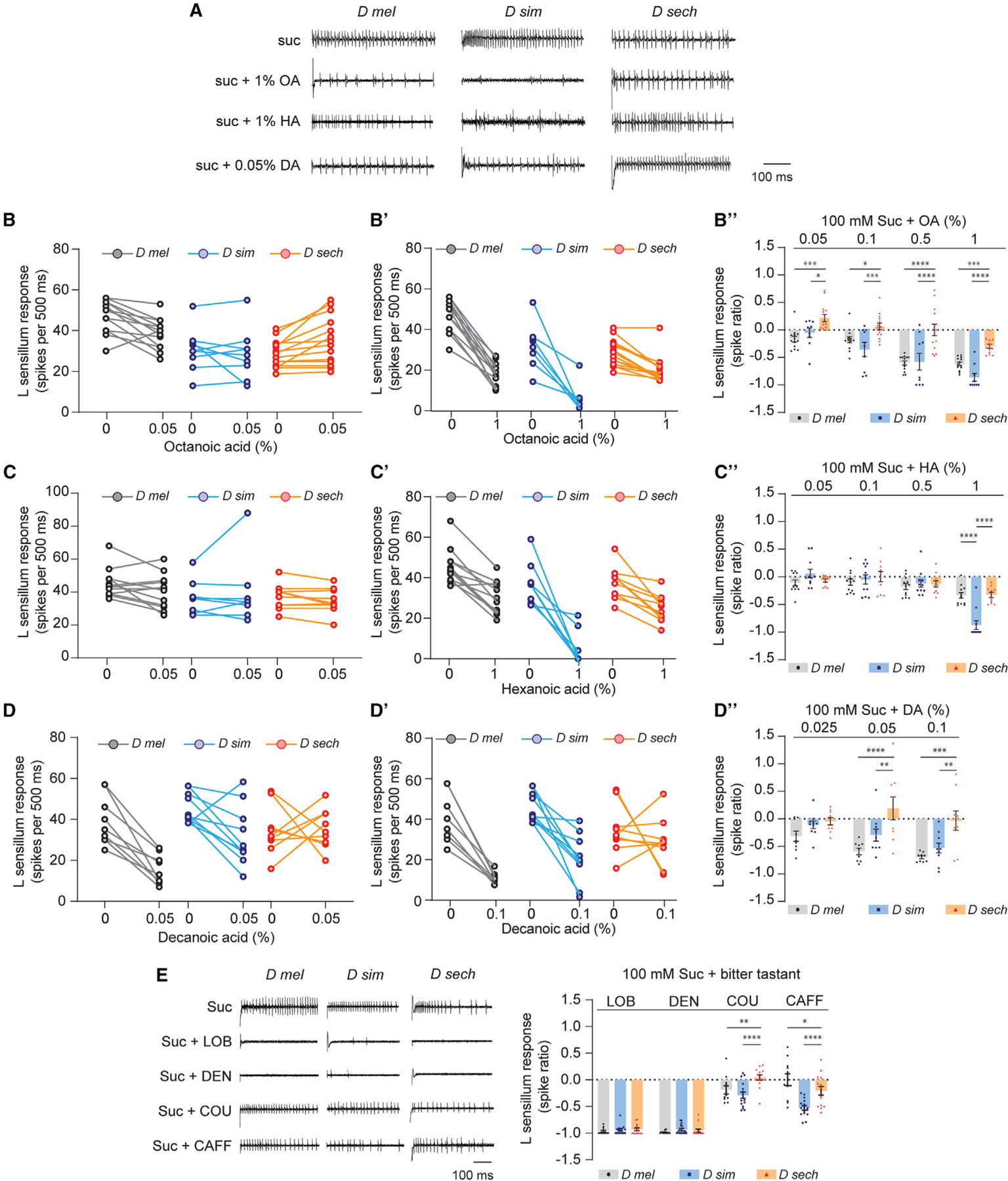
Noni FAs modulate sweet taste in a species-specific manner (A) Representative traces of recordings from L-type sensilla in named *Drosophila* species stimulated with 100 mM sucrose alone (suc) or in mixtures with octanoic acid (OA), hexanoic acid (HA), or decanoic acid (DA). Traces show the first 500-ms period upon stimulation with indicated tastant. (B and B”) Paired recordings from L-type sensilla of named *Drosophila* species with 100 mM sucrose alone (0) or in a mixture with OA (%). (B”) L-type sensillum responses to mixtures of 100 mM sucrose with varying concentrations of OA relative to 100 mM sucrose alone (depicted as spike ratios). n = 13 (*D mel*), n = 9 (*D sim*), n = 14 (*D sech*) from five flies. (C and C’) Paired recordings from L-type sensilla of named *Drosophila* species with 100 mM sucrose alone (0) or in a mixture with HA (%). (C”) L-type sensillum responses, calculated as in (B”), to mixtures of 100 mM sucrose with varying concentrations of HA relative to 100 mM sucrose alone. n = 12 (*D mel*), n=9 (*D sim*), n = 10 (*D sech*) from four flies. (D and D’) Paired recordings from L-type sensilla of named *Drosophila* species with 100 mM sucrose alone (0) or in a mixture with DA (%). (D”) L-type sensillum responses, calculated as in (B”), to mixtures of 100 mM sucrose with varying concentrations of DA relative to 100 mM sucrose alone. n = 9 (*D mel*), n = 10 (*D sim*), n = 9 (*D sech*) from four flies. (E) Representative traces and L-type sensillum responses, calculated as in (B”), to mixtures of 100 mM sucrose with bitter tastants lobeline (LOB, 10 mM), denatonium (DEN, 10 mM), coumarin (COU, 3 mM), and caffeine (CAFF, 10 mM), relative to 100 mM sucrose alone. n = 9 from four to five flies for each tastant-species combination. Data in (B”), (C”), (D”), and (E) were analyzed using two-way ANOVA for repeated measures with Tukey’s post hoc multiple comparisons test, *p < 0.05, **p < 0.01, ***p < 0.001, ****p < 0.0001. Error bars represent SEM. See also Figure S5.

In *D. melanogaster* and *D. simulans*, but not in *D. sechellia*, we observed significant reduction of sucrose-evoked firing activity in the presence of the highest concentrations of all three noni FAs (Figures 4A–4D). With OA and DA, a reduction of sucrose response was also observed with the lowest concentration that was tested. Since the baseline response to 100 mM sucrose varied across species, we quantified inhibition by calculating the ratio of spiking activity in response to the mixture to that of sucrose alone, which revealed differences in the threshold concentrations for this effect (Figures 4B’–4D’). Overall, DA > OA > HA in terms of strength of inhibition in the generalist species. Dose-dependent relationships were similar in *D. melanogaster* and *D. simulans*, with the exception that HA evoked stronger inhibition in the latter (Figure 4C’). Moreover, the inhibitory effects of FAs could be overcome by titrating the amount of sucrose (Figure S5), ruling out a scenario in which FAs have a general deleterious effect on neuronal function.

Notably, the action of noni FAs was markedly different on sugar-evoked activity in *D. sechellia*. At concentrations that were inhibitory in the generalist species, we found less inhibition in *D. sechellia* (Figures 4B’–4D’). Thus, loss of sweet taste inhibition may contribute to noni FA preference in *D. sechellia*.

We next asked whether the observed differences in inhibition are specific to noni FAs, or whether there is a broader alteration of the sweet taste neuron’s sensitivity to aversive tastants. We took recordings from L-type sensilla using mixtures of sucrose with bitter compounds, since many of these have been found to inhibit sweet-sensing neurons in *D. melanogaster*.^46,57–61^ Of the four tastants we selected, lobeline and denatonium exhibited strong inhibitory effects in *D. melanogaster*, whereas coumarin and caffeine did so weakly if at all (Figure 4E). We found no differences in the effect of lobeline and denatonium across the three species, indicating that the *D. sechellia* sweet-sensing neuron is capable of being inhibited by these compounds. Interestingly, coumarin and caffeine had stronger effects on sweet-sensing activity in *D. simulans*. We also observed smaller differences in the action of these compounds between *D. sechellia* and *D. melanogaster*. Overall, these results are consistent with the idea of FA-specific differences in patterns of sweet neuron inhibition across the three species.

### Noni FAs modulate sugar feeding preference in a species-specific manner

**Figure 5.**
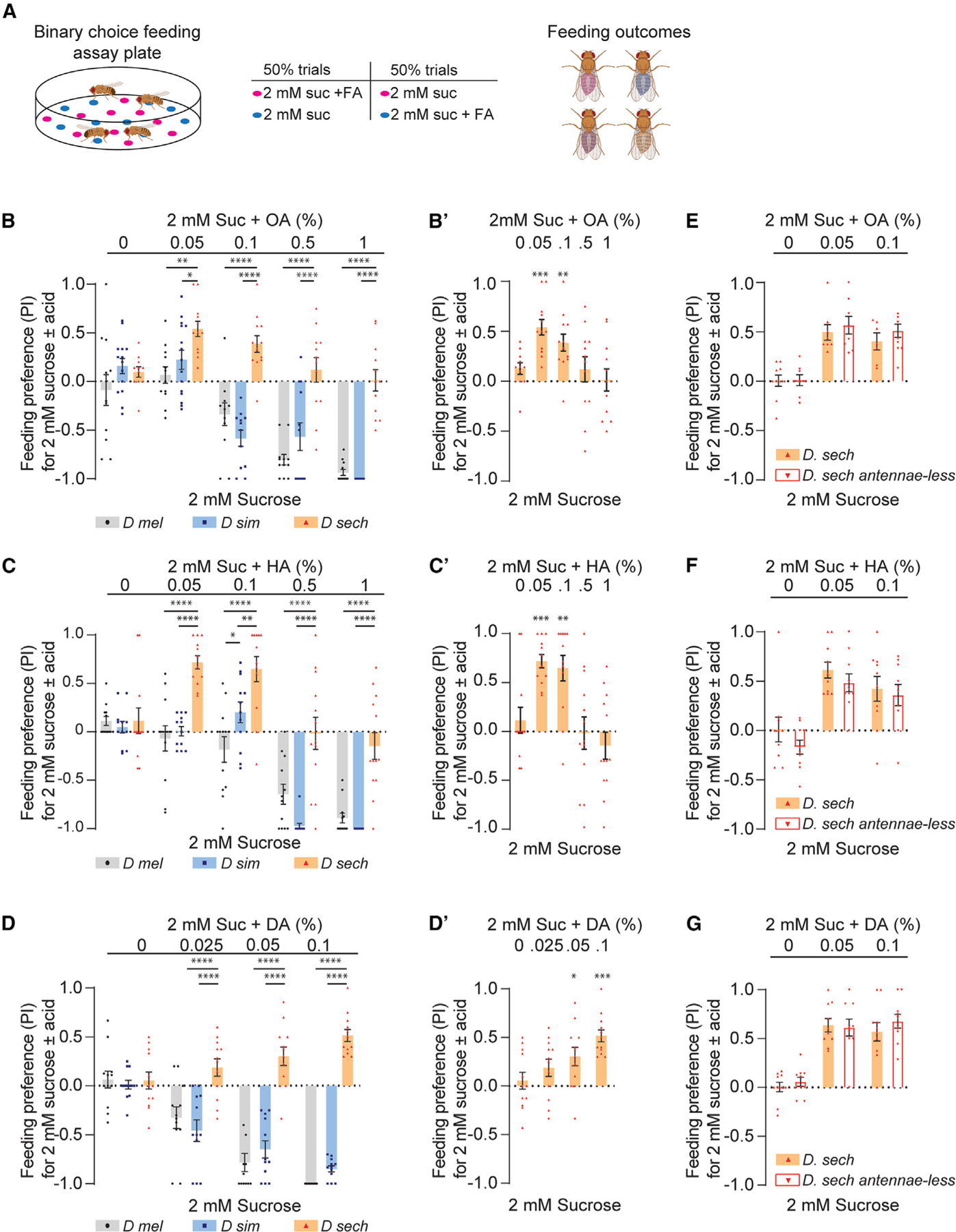
Noni FAs modulate sugar preference in a species-specific manner (A) Diagram of binary feeding assay used for (B)–(G) and outcomes. Created with BioRender.com. (B) Feeding preference of named species to 2 mM sucrose mixed with varying concentrations of octanoic acid (OA) tested against 2 mM sucrose alone. n = 12. (B’) *D. sechellia* results from (B), with preference index (PI) for each sucrose-OA mixture tested for significance from zero. (C) Feeding preference of named species to 2 mM sucrose mixed with varying concentrations of hexanoic acid (HA) tested against 2 mM sucrose alone. n = 12. (C’) *D. sechellia* results from (C), with preference index (PI) for each sucrose-HA mixture tested for significance from zero. (D) Feeding preference of named species to 2 mM sucrose mixed with varying concentrations of decanoic acid (DA) tested against 2 mM sucrose alone. n = 12. (D’) *D. sechellia* results from (D), with preference index (PI) for each sucrose-DA mixture tested for significance from zero. (E–G) Feeding preference of *D. sechellia*, tested intact (*D sech*) or with antennae surgically removed (*D sech antennae-less*), for 2 mM sucrose mixed with varying concentrations of OA (E), HA (F), or DA (G) tested against 2 mM sucrose alone. n = 9 (OA), n = 10 (HA), n = 10 (DA). Data in (B)–(G) were analyzed using two-way ANOVA with Tukey’s post hoc multiple comparisons test. Data in (B’)–(D’) were tested for significance from hypothetical mean 0 using the Wilcoxon test. For all graphs, *p < 0.05, **p < 0.01, ***p < 0.001, ****p < 0.0001. Error bars represent SEM.

We next examined how noni FAs influence feeding responses to sugar, using a series of binary assays in which both enhancement and suppression of feeding preference could be measured. In these experiments, both tastant alternatives contained the same concentration of sucrose (2 mM), and one also included a noni FA (Figure 5A). Each of the noni FAs were tested across a range of concentrations as noted above. In these assays, a PI (preference index) greater than zero would indicate a positive behavioral valence for the noni FA mixture, and conversely a value less than zero would indicate a negative behavioral valence.

The two generalist species showed little to no preference for any of the mixtures with noni FAs at lower concentrations and strong avoidance as the concentrations increased (Figures 5B–5D). Conversely, *D. sechellia* displayed preference for the sucrose-noni FA mixture across multiple concentrations (PI is significantly different from zero), suggesting that the noni compound increased the appetitive value of the mixture (Figures 5B–5D and 5B’–5D’). To determine whether the attraction is dependent on olfactory input, we surgically removed the antennae, which house sensilla that show heightened sensitivity to noni FAs and related volatiles.^32,37,38^ We found that *D. sechellia*’s feeding preference for sucrose-noni FA mixtures was not altered by removal of the main olfactory organ (Figures 5E–5G). Although we cannot rule out possible involvement of the maxillary palps, these results are consistent with a primary role for taste function in driving feeding outcomes to sucrose-FA mixtures.

### *Ir47a* mutants of *D. melanogaster* exhibit *D. Sechellia*-like responses to mixtures with low noni FAs

A previous study reported that Ir76b, a broadly expressed ionotropic receptor, can modulate the sensitivity of sweet-sensing taste neurons.^62^ In *D. melanogaster* flies lacking *Ir76b*, the responses to mixtures of sucrose and acetic acid were abnormally high. We wondered if *Ir76b* might similarly be involved in lowering the response of sweet-sensing neurons in the presence of noni FAs.

**Figure 6.**
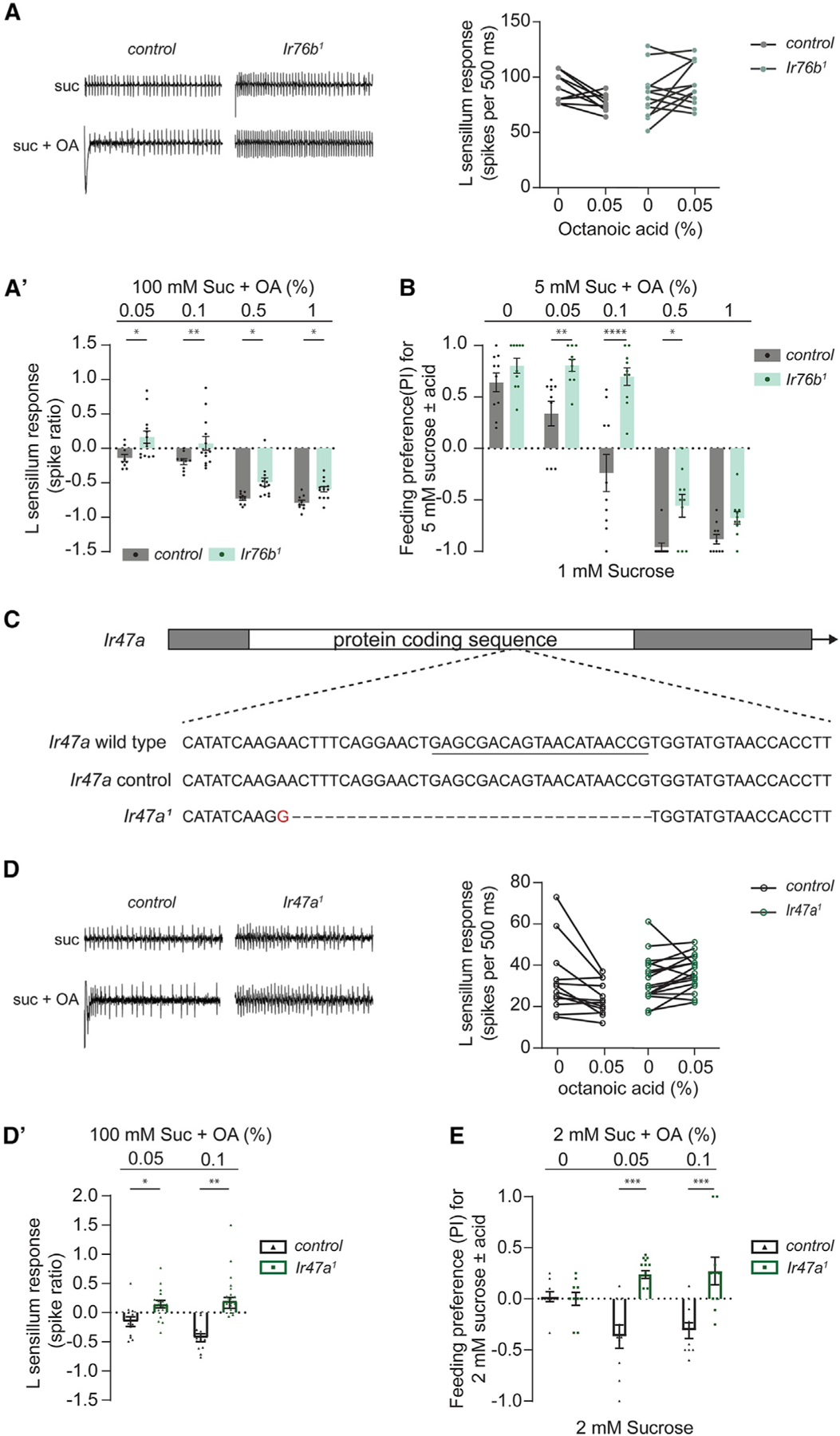
*D. melanogaster Ir* mutants exhibit *D. sechellia*-like responses to low FA-sucrose mixtures (A) Representative traces (left) and paired recordings (right) from L-type sensilla of *D. melanogaster w*^1118^ (control) and *Ir76b*^1^ flies with 100 mM sucrose alone (0) or in a mixture with 0.05% octanoic acid (0.05). (A’) L-type sensillum responses to mixtures of 100 mM sucrose with varying concentrations of octanoic acid (OA) relative to 100 mM sucrose alone (depicted as spike ratio); data from (A) are included in this graph. Genotypes are as in (A). n = 9–13 from four flies. (B) Feeding preference of *w*^1118^ (control) and *Ir76b*^1^ flies for 5 mM sucrose mixed with varying concentrations of octanoic acid (OA), tested against 1 mM sucrose. n = 10. (C) Diagram of *Ir47a* depicting the lesion in the *Ir47a*^1^ allele, generated using CRISPR-Cas9. The sequence targeted by the sgRNA is underlined. (D) Representative traces (left) and paired recordings (right) from L-type sensilla of *D. melanogaster w*^1118^ (control) and *Ir47a*^1^ flies with 100 mM sucrose alone (0) or in a mixture with 0.05% octanoic acid (0.05). (D’) L-type sensillum responses to mixtures of 100 mM sucrose with varying concentrations of octanoic acid (OA) relative to 100 mM sucrose alone (depicted as spike ratio); data from (D) are included in this graph. Genotypes are as in (D). n = 12–17 from 5 to 6 flies. (E) Feeding preference of *w*^1118^ (control) and *Ir47a*^1^ flies for 2 mM sucrose mixed with varying concentrations of octanoic acid (OA), tested against 2 mM sucrose. n = 10. Data in (B) and (E) were analyzed using two-way ANOVA with Sidak’s post hoc test for multiple comparisons; data in (A’) and (D’) were analyzed with two-way ANOVA for repeated measures with Sidak’s post hoc multiple comparisons test. *p < 0.05, **p < 0.01, ***p < 0.001, ****p < 0.0001. Error bars represent SEM.

We recorded from L-type sensilla in *D. melanogaster Ir76b* mutants using mixtures of sucrose with OA. Inclusion of OA at concentrations of 0.05% and 0.1% lowered the response in control flies not in *Ir76b* mutants (Figures 6A and 6A’). At OA concentrations of 0.5% and 1%, sugar response inhibition was slightly reduced but still present in the mutant flies (Figure 6A’). Consistent with the electrophysiological analysis, we found that *Ir76b* mutants showed higher feeding preference for sucrose-OA mixtures, particularly with the lower concentrations of OA, than control flies (Figure 6B). Our results invoke an *Ir76b*-dependent mechanism that lowers sucrose response in the presence of FAs along with an additional *Ir76b*-independent mechanism that contributes to sugar response inhibition at higher FA concentrations. Further, we hypothesized that the *Ir76b*-dependent pathway may be altered in *D. sechellia*.

The genome of *D. melanogaster* encodes 63 Ir receptors, a number of which are expressed in taste neurons.^42,63–65^ A comparison of the Ir repertoires in the three drosophilid species used in this study shows that 13 are selectively pseudogenized in *D. sechellia*.^66^ Among these, *Ir47a* emerged as a candidate of interest because its expression was previously mapped to sweet-sensing neurons.^63^ To test whether removal of *Ir47a* function in *D. melanogaster* would phenocopy *D. sechellia* in terms of higher responses to mixtures of sucrose and low concentrations of OA, we used CRISPR-Cas9 to generate a mutant allele of *Ir47a* (Figure 6C) and recorded from L-type sensilla in flies lacking *Ir47a* function. We found that in *Ir47a* mutants, mixtures of sucrose with OA (0.05% or 0.1%) did indeed evoke more spikes than sucrose alone (Figures 6D and 6D’). Binary choice assays (Figure 6E) suggest a valence switch in *Ir47a* mutants at the tested concentrations of OA—the mutants showed a positive mean preference for the sucrose-OA mixtures, notably at concentrations that evoked a negative mean preference in controls. Overall, these results raise the possibility that loss of *Ir47a* in *D. sechellia* contributes to its altered pattern of feeding preference for noni.

## DISCUSSION

**Figure 7.**
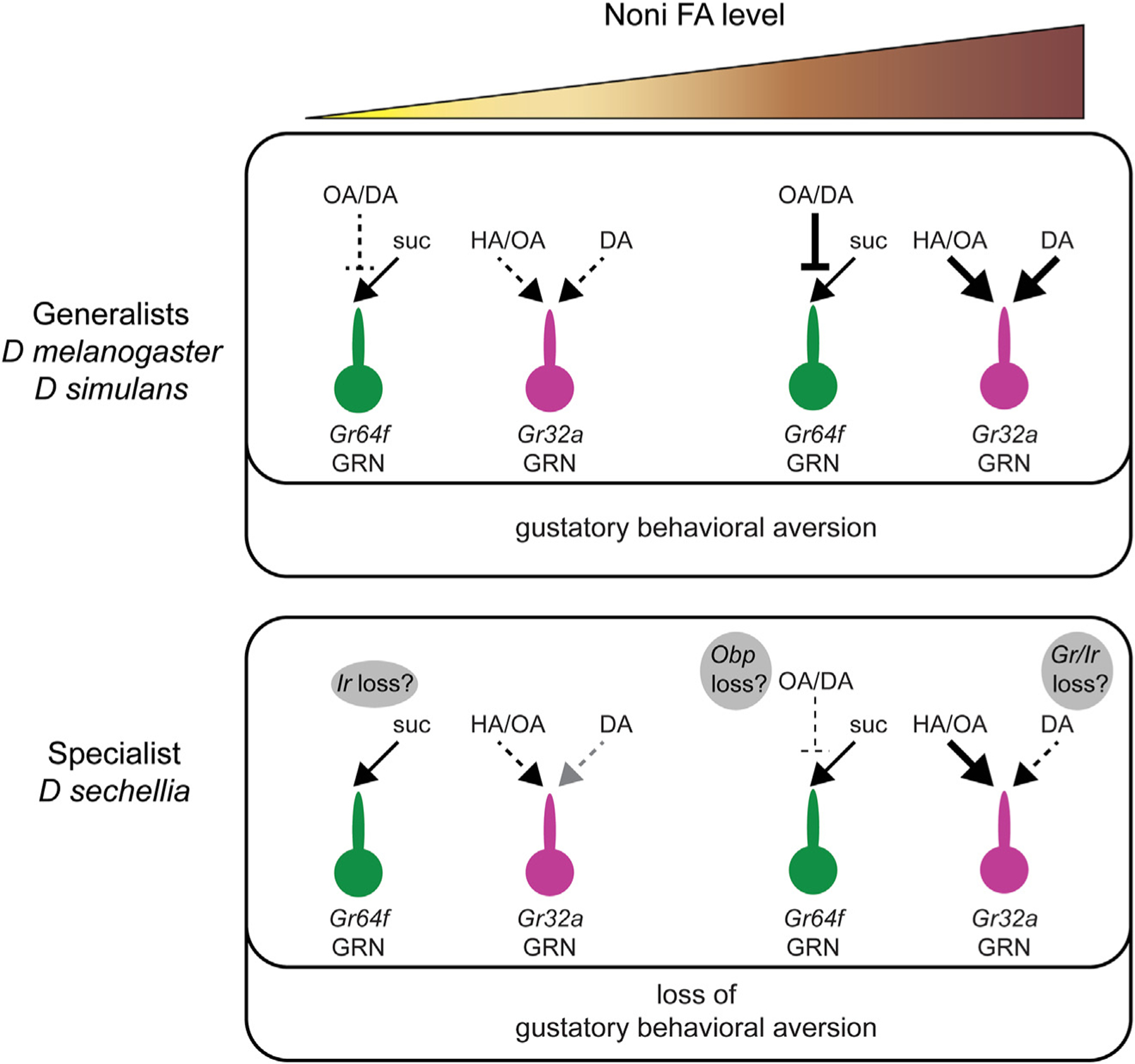
Proposed model for loss of noni taste aversion in *D. sechellia* Diagram summarizing proposed cellular and molecular mechanisms underlying observed differences in gustatory aversion to noni FAs between generalist drosophilids and *D. sechellia*. The action of noni FAs in mixtures with sucrose is depicted for *Gr64f* GRNs; not included here is the activation of *Gr64f* neurons with pure FAs. Molecular pathways that are predicted to have loss of function variants in *D. sechellia* are indicated in the gray bubbles. Noni FAs is used as a generic term; HA, hexanoic acid; OA, octanoic acid; DA, decanoic acid.

In this study we investigate cellular and behavioral responses of three closely related species—*D. sechellia*, *D. simulans*, and *D. melanogaster*—to uncover the gustatory basis of *D. sechellia’s* specialization on noni fruit. Our results suggest that gustatory adaptation is complex, involving changes in multiple response features of taste neurons to alter the specialist’s preference for noni fruit (Figure 7). Specifically, we find that FAs activate bitter taste neurons and inhibit sugar responses in sweet taste neurons, with FA- and species-specific patterns matching the switch in behavior between the generalists and *D. sechellia*. Genetic analysis in *D. melanogaster* predicts chemosensory variants in *D. sechellia* that alleviate FA-mediated suppression of sugar response and reduce FA-mediated activation of bitter taste neurons. Furthermore, our study does not exclude the possibility of gain-of-function alterations in chemoreceptors such as Ir56d that mediate taste attraction to FAs.^40–42^ Future work could determine whether similar adaptations are found in *D. yakuba mayottensis*, which has independently specialized on noni fruit.^67,68^

Identification of the genetic basis of evolutionary variation in noni taste poses significant challenges. Nevertheless, an understanding of the neurophysiological differences in *D. sechellia* allows us to pinpoint a number of chemosensory genes that are predicted to be non-functional^66,69,70^ or that show reduced expression in the labellum of *D. sechellia*^35,52^ as candidates that merit further investigation. Our work presents clear hypotheses for roles of candidate *Gr*, *Ir*, and *Obp* genes in the evolution of noni FA taste in *D. sechellia* (Figure 7). It is also possible that peripheral adaptation is accompanied by anatomical and/or functional changes in gustatory circuits, but this is far more difficult to address within the confines of our current tools and understanding.

Large chemosensory gene families in insects provide evolutionary forces with many substrates on which to act and alter peripheral function. Comparison of drosophilid species show rapid evolution of receptor genes in the genome^35,69,70^ as well as changes in gene expression across species.^4,52^ Comparative analyses of chemosensory functions have also begun to reveal extensive plasticity in the periphery.^4,32,37,38,52,71,72^ A notable example is the olfactory system of *D. sechellia*, in which Or22a and Ir75b variants with increased sensitivity to noni volatiles are expressed in greater numbers of olfactory neurons,^32,37,38^ which likely contribute to the increased attraction of this species to noni fruit, and also gate close-range taste-driven oviposition preference.^73^ Another example is that of the fruit pest, *D. suzukii*, which has substantially reduced expression of bitter *Gr* genes and shows a corresponding loss of sensitivity to a broad range of bitter tastants, which correlates with its shift in oviposition preference for ripe fruit compared to overripe fruit.^4^

From this study, *Ir* and *Obp* genes emerge as candidates for evolution of sweet-sensing neuron activity in response to noni FA-sugar mixtures. In the presence of sucrose, we uncovered inhibitory effects of noni FAs on sweet-sensing neurons, which appear to depend, at least in part, on Ir76b/Ir47a and perhaps additional Irs. Given that *D. melanogaster Ir47a* mutants exhibit *D. sechellia*-like responses to low OA-sucrose mixtures, it would be interesting to restore *Ir47a* in *D. sechellia* to test whether the functional loss of this particular chemoreceptor is a contributing factor in its behavioral evolution. Another potential mechanism is one involving Obps. One member of this family, *Obp49a*, is known to be required for inhibition of sweet taste neurons by bitter tastants and is downregulated in *D. sechellia*.^46,74^ *Obp56e* and *Obp57d/e*, which alter perception of noni FAs,^33–35^ may also be involved via their action on sweet-sensing taste neurons. Alternatively, additional candidates may be tested on the basis of comparative genome or expression analyses. A few *Obps* are appealing because they exhibit evolutionary rate differences between specialist and generalist species in the melanogaster subgroup^75^ or are differentially expressed in the proboscis.^74^

Similarly, specific *Gr* genes are candidates for roles in noni FA-mediated bitter-sensing neuron activity. Our observation that *Gr33a* mutants of *D. melanogaster* show reduced aversion to noni FAs is consistent with a model in which a *Gr* variant in *D. sechellia* alters its bitter response to DA and thereby contributes to its noni preference. This fits with the rapid loss of Grs in *D. sechellia—*13 of 73 Grs found in *D. simulans* are predicted to be lost in *D. sechellia*, and at least some of them were expected to be involved in sensing compounds that deter the generalist sibling from noni.^70^ Additionally, a number of bitter *Grs* were found to have signals of soft selective sweeps in *D. yakuba mayottensis*, supporting the involvement of the *Gr* family in behavioral adaptation in this independent model of noni specialization.^76^ Nevertheless, given the extent of *Gr* variation, it was somewhat of a surprise that *D. sechellia* exhibited fairly robust bitter taste responses to HA and OA. At least three different reasons might account for this. One, our study may have missed features of FA responses since we did not record from labellar I-type hairs or any tarsal sensilla, which could be differentially tuned to noni FAs in the different species. Although responses from Sa- and Sb-type sensilla appeared similar and were pooled for analysis, it is possible that small differences between these subtypes could be obscured by this strategy. With tip recordings, we also could not measure OFF responses, compounds,^77^ consistent with the idea that *D. sechellia* has lost *Gr* genes that recognize bitter tastants that it is no longer exposed to.^70^ Similarly, a broad loss of bitter sensitivity was found to correlate with the shift in oviposition substrate preference observed from *D. melanogaster* to *D. suzukii*.^4^ Finally, at least two instances of pseudo-pseudogenes are described in drosophilids.^72,78^ Perhaps some of the Grs that are considered non-functional in *D. sechellia* may not be so. Future genetic experiments with *D. sechellia* will be invaluable to provide insight into the adaptive reported as elevations in calcium activity upon removal of certain organic and fatty acids, and it is conceivable that these vary across species.^54,55^ Alternatively, *Gr* losses may be unrelated to FA taste evolution. A recent study found that losses of *Gr28b.a* and *Gr39a.a* in *D. melanogaster* phenocopy *D. sechellia* in terms of reducing feeding aversion to other bitter roles of any candidate genes that emerge from initial tests in *D. melanogaster*.

Further analysis of gustatory variation might prove of immense value in studying the evolution of host specialization and speciation. One proposed model is that loss of behavioral aversion to noni, which appears to be significantly dependent on gustation, preceded selection of physiological resistance and mechanisms to increase attraction.^11,48^ Analysis of the three chosen species did not yield a clear hypothesis for a temporal sequence of the observed gustatory adaptations. Since *D. simulans* presented heightened noni aversion compared with *D. melanogaster*, questions remain about the ancestral state of noni FA taste. A broader comparative approach with additional drosophilid species, including more of the simulans clade, may help to determine the evolutionary history of distinct gustatory mechanisms and their potential contributions to behavior across the phylogeny.

### Limitations of the study

We did not observe sweet taste neuron activity upon stimulation with FAs alone, in contrast with previous findings of robust FA-evoked calcium transients in *Gr64f*^+^ sweet-sensing neurons. This could be due to differences in sampling properties and temporal resolution between calcium imaging in the presynaptic termini and tip recordings from the dendrites. A more plausible explanation is that our recordings were not conducted from the relevant labellar sensilla. A recent study found HA-evoked firing rates of >5 spikes per second only in L4 and L6 sensilla,^56^ while we largely focused on L-type sensilla #7–9. Therefore, our study does not investigate potential differences in FA-induced activation of sweet-sensing neurons between the three species. It is worth noting that variation in noni FA taste may also exist in other organs such as the tarsi, where FA-responsive neurons are known to reside.^41^

## STAR★METHODS

### RESOURCE AVAILABILITY

#### Lead contact

Further information and requests for resources and reagents should be directed to and will be fulfilled by the lead contact, Anupama Dahanukar, anupama.dahanukar@ucr.edu.

#### Materials availability

New plasmids and fly lines generated in this study are available upon request. Requests should be directed to and will be fulfilled by the lead contact.

#### Data and code availability

- All original data have been deposited at Mendeley Data and are publicly available as of the date of publication. DOIs are listed in the key resources table.
- The paper does not report original code.
- Any additional information required to reanalyze the data reported in this study is available from the lead contact upon request.

### EXPERIMENTAL MODEL AND STUDY PARTICIPANT DETAILS

#### *Drosophila* stocks

Flies were raised on standard cornmeal-dextrose diet at 25°C and 50% relative humidity in a 12:12 light:dark cycle. For the diet, yellow cornmeal (57020, Quaker), dextrose (G8270, Sigma), inactive dry yeast (75570, Lynside), propionic acid (402907, Sigma) and tegosept (20–258, Apex chemical and reagent) were used. *D. melanogaster* flies were red-eyed *Canton-S*. Other *D. melanogaster* genotypes were obtained from the Bloomington *Drosophila* Stock Center: *UAS-Kir2.1* (BL91802)*, Gr64f-Gal4* (BL27883)*, Gr32a-Gal4* (BL57622), *Gr33a*^1^ (BL31427), *Ir56d*^1^ (BL81249), *Ir76b*^1^ (BL51309), *orco*^1^ (BL23129), *vas-Cas9* (BL# 51324), *w*^1118^ (BL5905). *wCS* is *w*^1118^ backcrossed to Canton-S. *D. simulans* (w[501] 14021–0251.195) and *D. sechellia* (14021–0248.25) were obtained from the *Drosophila* Species Stock Center. Δ*Gr64a-f* flies were generously shared by Dr. Seok Jun Moon. *Ir47a*^1^ mutants were generated in the laboratory for this study.

### METHOD DETAILS

#### Generation of *Ir47a*^*1*^ mutant

The *Ir47a*^1^ mutant was generated using CRISPR/Cas9. Synthetic oligos (sense 5’-CTTCGAGCGACAGTAACATAACCG and antisense 5’-AAACCGGTTATGTTACTGTCGCTC were annealed and cloned into the pBS-U6-BbsI-chiRNA (#45946, Addgene) restricted with BbsI. The U6-Ir47a-chiRNA cassette was removed using KpnI and EcoRI digestion and cloned into similarly cut pattB. An injection service (Bestgene, Inc) was used to transform y,w; attP40 embryos with the resulting pattB[U6-Ir47a-chiRNA] vector using the site-directed phiC31 integrase system. *vas-Cas9* females were mated with attP40[Ir47a-chiRNA] males and the resulting attP40[Ir47a-chiRNA]/+; vas-Cas9/+ females were mated to balancer males to generate isogenic lines. 8 lines exhibited indels at the CRISPR target site representing *Ir47a*^1^ and 2 other frameshift alleles and 5 in-frame deletion alleles.

#### Proboscis extension response assay

5–7 days old female flies were starved for 24–26 h on water-saturated Kimwipes. For experiments, individual flies were trapped in 20 μL pipette tips (1123–1710, USA Scientific) such that their heads were exposed. Flies were given water until satiated and then stimulated with wicks wetted with 30 mM sucrose. Flies that did not cease to drink water or that failed to respond to sucrose were discarded. Tastants were tested from low to high concentrations and water was given between tastants. Responses were scored as follows: full proboscis extension = 1, partial proboscis extension = 0.5, no proboscis extension = 0.

#### Binary choice feeding preference assay

Feeding preferences were measured using the Binary Choice Assay. Tastant solutions were prepared in water and tested for pH with indicator papers (4391–01, Baker-pHIX). pH was in the moderate to neutral range.

**Table:** 

pH values of tastant solutions prepared for behavior experiments.			
Concentration	OA	HA	DA
0.025%			7
0.05%	6.5	7	7
0.10%	6.5	6.5	5.5
0.50%	5	5	
1.00%	4.5	4	

Equal numbers of trials were performed with dyes swapped. To ensure sufficient participation 5–7 days old flies were wet-starved for 24–26 h prior to the experiment. Groups of 10 males and 10 females were then placed in tight-fit Petri dishes (351006, Falcon) containing 1% agarose droplets mixed with pink (0.5 mg/mL sulforhodamine) or blue (0.25 mg/mL indigocarmine) dyes and tastants. Food droplets were dispensed on the Petri plates using 0.5 mL repeating pipette tips (4751–0050, Tip One). Once flies were added, plates were kept in a dark humid chamber (25°C) for 2 h, after which the flies were frozen at −80°C for 30 min and scored for the color in their abdomens. Feeding preference was calculated as follows:

Preference Index (PI) = ((# flies fed on tastant 1) – (# flies fed on tastant 2))/(# flies fed on tastant 1 or tastant 2 or both) Trials with less than 50% participation were discarded.

#### Consumption assay

5–7 days old flies were wet-starved for 24–26 h. Groups of 10 males and 10 females were then placed in tight-fit Petri plates (Falcon 351006) containing 1% agar droplets mixed with pink (0.5 mg/mL sulforhodamine) or blue (0.25 mg/mL indigocarmine) dyes and tastants. Equal numbers of trials were performed with dyes swapped. Plates were kept in a dark humid chamber (25°C) for 2 h and then frozen at −80°C for 30 min. Female flies from each plate were first weighed and then their digestive tracts were dissected with forceps (#11252–20, Fine Science Tools) and placed in PCR tubes (AB-620, Thermo Scientific) with 5 μL of water. The tubes were vortexed and spun in a mini-centrifuge (C1413V-115 Kinetic Energy 26 J Galaxy Mini Centrifuge, VWR) for 30 s. Supernatant was collected using 10μL XL graduated pipette tips (1110–3800, USA Scientific) and the absorption was measured at 565 nm (for pink food) and 289 nm (for blue food) using a Nanodrop 2000c Spectrometer. Standard curves were generated for both dyes to calculate ingested food volumes from absorbance measurements.

#### Extracellular tip recordings

Extracellular recordings were performed from S- and L-type sensilla in the labellum using the tip recording method.^79^ Female flies aged 5–7 days were used for the recordings. A reference electrode, filled with Beadle-Ephrussi Ringer Solution (7.5 g NaCl, 0.35 g KCl, 0.279 g CaCl_2_.2H_2_0) was inserted into the dorsal thorax and into the labellum such that it immobilized the proboscis in a completely extended position. Tastants were dissolved in 30 mM tricholine citrate (TCC); the pH range for all tastant stimuli was 6.5–7.0. Recordings were obtained using a TasteProbe coupled with an IDAC-4 signal acquisition system and Autospike software (Syntech). Up to four sensilla of each type were recorded from a single fly, and at least four flies were tested for each stimulus*^−^ sensillum type combination. Species or genotypes to be compared within experiments were tested on the same day(s). Neuronal activity was quantified in the first 500-ms period following contact between the stimulus and the pore of the taste sensillum. Spike ratios were calculated as follows: (#spikes_100 mM sucrose + FA_ – #spikes_100 mM sucrose_)/(#spikes_100 mM sucrose_).

#### *In vivo* calcium imaging

3–5 days old mated female flies expressing UAS-GCaMP-R (GCaMP6.0 and mCherry) in *Gr66a* neurons were starved for 24 h prior to imaging, as previously described.^40,50^ Flies were anesthetized on ice and then placed inside of a cut 200 μL pipette tip (#50101182, Fisher Scientific) so that their head and proboscis were accessible, but their body and tarsi were restrained. Using forceps (#11252–20, Fine Science Tools), the proboscis was manually extended and a small amount of dental glue (#595953WW, Ivoclar Vivadent Inc) was applied between the labium and the edge of the pipette tip, ensuring the same position throughout the experiment. Next, both antennae were removed. A small hole ~1 mm in diameter was cut into a 1 cm^2^ piece of aluminum foil and then fixed to the fly using dental glue, creating a sealed window of exposed cuticle. Artificial hemolymph (140 mM NaCl, 2 mM KCl, 4.5 mM MgCl2, 1.5 mM CaCl2, and 5 mM HEPES-NaOH with pH = 7.1) was applied to the window and then the cuticle and connective tissue were dissected using forceps and a 27 g hypodermic needle (#14-840-82, Fisher Scientific) to expose the SEZ. Mounted flies were placed on a Nikon A1R confocal microscope and then imaged using a 20X water-dipping objective lens. The pinhole was opened to allow a thicker optical section to be monitored. *Gr66a* neurons were simultaneously excited with wavelengths of 488 nm (FITC) and 561 nm (TRITC). All recordings were taken at 4Hz with 256 resolution. Tastants were applied to the proboscis with a wick, which was operated using a micromanipulator (Narishige International USA, Inc). For initial measurements of ON responses, tastants were applied for ~2 s. For ON and OFF responses, tastants were applied to the proboscis for ~10 s. For analysis, regions of interest were drawn manually around the *Gr66a* projections. For each frame, the mean fluorescence intensity for FITC and TRITC was subtracted from the background mean fluorescence intensity. Then, the fluorescence ratio of GCaMP6.0 to mCherry was calculated. Next, baseline fluorescence was calculated as the average fluorescence ratio of the first 5 frames, 10 s prior to tastant application. For each frame, the % change in fluorescence (%ΔF/F) was then calculated as: (peak fluorescence ratio - baseline fluorescence ratio)/baseline fluorescence ratio * 100. Average fluorescence traces were created by taking the average and standard error of %ΔF/F for each recording of a specific tastant.

### QUANTIFICATION AND STATISTICAL ANALYSES

#### Statistical analyses

Sample sizes for all experiments were determined from previous literature. All statistical analyses were performed using Graphpad Prism software. Data from PER experiments were analyzed using Friedman Test with Dunn’s *post hoc* analysis. Data from electrophysiology experiments were analyzed using two-way ANOVA with repeated measures for pairwise comparisons or Mann Whitney test. Data from binary choice assays were analyzed using two-way ANOVA with pairwise comparisons or *t*-tests with Welch’s correction. The results of consumption assays were compared using *t*-tests with Welch’s correction. Calcium responses were compared using one-way ANOVA. Details, including the statistical tests used and the exact values of *n* for each experiment are provided in the corresponding figure legends. In all graphs, error bars depict s.e.m. Results of all statistical analyses are included in Mendeley data (https://doi.org/10.17632/srrd35bxcm.1).

## Supplementary Material

1

## Figures and Tables

**Table:** KEY RESOURCES TABLE

REAGENT or RESOURCE	SOURCE	IDENTIFIER
Chemicals, peptides, and recombinant proteins		

Caffeine	Sigma-Aldrich	C8960
Coumarin	Sigma-Aldrich	C4261
Decanoic acid	Sigma-Aldrich	C1875
Hexanoic acid	Sigma-Aldrich	153745
Indigocarmine	Sigma-Aldrich	18130
Lobeline	Sigma-Aldrich	141879
Sucrose	Sigma-Aldrich	S7903
Sulforhodamine	Sigma-Aldrich	230162
Tricholine citrate	Sigma-Aldrich	T0252
Octanoic acid	Sigma-Aldrich	C2875
Quinine	Sigma-Aldrich	22630

Deposited data		

Original data and results of statistical analyses	This paper, Mendeley data	https://doi.org/10.17632/srrd35bxcm.1

Experimental models: Organisms/strains		

*D. melanogaster*, Canton-S		N/A
*D. sechellia*	*Drosophila* Species Stock Center	14021-0248.25
*D. simulans, w[501]*	*Drosophila* Species Stock Center	14021-0251.195
*D. melanogaster: Gr32a-GAL4*	Bloomington *Drosophila* Stock Center	57622
*D. melanogaster: Gr33a* ^1^	Bloomington *Drosophila* Stock Center	31427
*D. melanogaster:* Δ*Gr64a-f*	Dr. Seok Jun Moon’s laboratory^49^	N/A
*D. melanogaster: Gr64f-GAL4*	Bloomington *Drosophila* Stock Center	27883
*D. melanogaster: Ir47a* ^1^	This paper, Figure 6	N/A
*D. melanogaster: Ir56d* ^1^	Bloomington *Drosophila* Stock Center	81249
*D. melanogaster: Ir76b* ^1^	Bloomington *Drosophila* Stock Center	51309
*D. melanogaster: orco* ^1^	Bloomington *Drosophila* Stock Center	23129
*D. melanogaster: UAS-Kir2.1*	Bloomington *Drosophila* Stock Center	91802
*D. melanogaster: vas-Cas9*	Bloomington *Drosophila* Stock Center	51324
*D. melanogaster: w* ^1118^	Bloomington *Drosophila* Stock Center	5905
Oligonucleotides		
5’-CTTCGAGCGACAGTAACATAACCG	Integrated DNA Technologies	N/A
5’-AAACCGGTTATGTTACTGTCGCTC	Integrated DNA Technologies	N/A

Recombinant DNA		

pBS-U6-BbsI-chiRNA	Addgene	45946
pattB[U6-Ir47a-chiRNA]	This paper, Figure 6	N/A

## Highlights

- *D. melanogaster* and *D. simulans*, but not *D. sechellia*, avoid feeding on noni acids
- Noni fatty acids (FAs) activate bitter taste neurons and inhibit sweet taste neurons
- *D. sechellia* has FA-specific changes in bitter activation and sweet inhibition
- Variations in *Gr* and *Ir* genes are predicted to alter noni FA taste in *D. sechellia*

## References

[1] Jacquin-Joly E, and Merlin C (2004). Insect olfactory receptors: contributions of molecular biology to chemical ecology. J. Chem. Ecol 30, 2359–2397. 10.1007/s10886-004-7941-3. 15724962

[2] Anholt RRH (2020). Chemosensation and evolution of Drosophila host plant selection. iScience 23, 100799. 10.1016/j.isci.2019.100799. 31923648 PMC6951304

[3] Lizana P, Mutis A, Quiroz A, and Venthur H (2022). Insights into chemosensory proteins from non-model Insects: Advances and perspectives in the context of pest management. Front. Physiol 13, 924750. 10.3389/fphys.2022.924750. 36072856 PMC9441497

[4] Dweck HK, Talross GJ, Wang W, and Carlson JR (2021). Evolutionary shifts in taste coding in the fruit pest Drosophila suzukii. Elife 10, e64317. 10.7554/eLife.64317. 33616529 PMC7899650

[5] Linz J, Baschwitz A, Strutz A, Dweck HKM, Sachse S, Hansson BS, and Stensmyr MC (2013). Host plant-driven sensory specialization in Drosophila erecta. Proc. Biol. Sci 280, 20130626. 10.1098/rspb.2013.0626. 23595274 PMC3652467

[6] Missbach C, Dweck HK, Vogel H, Vilcinskas A, Stensmyr MC, Hansson BS, and Grosse-Wilde E (2014). Evolution of insect olfactory receptors. Elife 3, e02115. 10.7554/eLife.02115. 24670956 PMC3966513

[7] van Schooten B, Melé ndez-Rosa J, Van Belleghem SM, Jiggins CD, Tan JD, McMillan WO, and Papa R (2020). Divergence of chemosensing during the early stages of speciation. Proc. Natl. Acad. Sci. USA 117, 16438–16447. 10.1073/pnas.1921318117. 32601213 PMC7371972

[8] Auer TO, Shahandeh MP, and Benton R (2021). Drosophila sechellia: A genetic model for behavioral evolution and neuroecology. Annu. Rev. Genet 55, 527–554. 10.1146/annurev-genet-071719-020719. 34530638

[9] Schä pers A, Carlsson MA, Gamberale-Stille G, and Janz N (2015). The role of olfactory cues for the search behavior of a specialist and generalist butterfly. J. Insect Behav 28, 77–87.

[10] Lachaise D, and Silvain JF (2004). How two Afrotropical endemics made two cosmopolitan human commensals: the Drosophila melanogaster-D. simulans palaeogeographic riddle. Genetica 120, 17–39. 10.1023/b:gene.0000017627.27537.ef. 15088644

[11] Jones CD (2005). The genetics of adaptation in Drosophila sechellia. Genetica 123, 137–145. 10.1007/s10709-004-2728-6. 15881686

[12] R’Kha S, Capy P, and David JR (1991). Host-plant specialization in the Drosophila melanogaster species complex: a physiological, behavioral, and genetical analysis. Proc. Natl. Acad. Sci. USA 88, 1835–1839. 10.1073/pnas.88.5.1835. 1900368 PMC51120

[13] Sauer JD (1960). Coastal Plant Geography of Mauritius (LOUISIANA STATE UNIV BATON ROUGE COASTAL STUDIES INST)

[14] Robertson SA (1989). Flowering Plants of Seychelles (Royal Botanic Gardens)

[15] Hey J, and Kliman RM (1993). Population genetics and phylogenetics of DNA sequence variation at multiple loci within the Drosophila melanogaster species complex. Mol. Biol. Evol 10, 804–822. 10.1093/oxfordjournals.molbev.a040044. 8355601

[16] Kliman RM, Andolfatto P, Coyne JA, Depaulis F, Kreitman M, Berry AJ, McCarter J, Wakeley J, and Hey J (2000). The population genetics of the origin and divergence of the Drosophila simulans complex species. Genetics 156, 1913–1931. 10.1093/genetics/156.4.1913. 11102384 PMC1461354

[17] Legal L, Chappe B, and Jallon JM (1994). Molecular basis of Morinda citrifolia (L.): Toxicity on Drosophila. J. Chem. Ecol 20, 1931–1943. 10.1007/BF02066234. 24242720

[18] Amlou M, Moreteau B, and David JR (1998). Genetic analysis of Drosophila sechellia specialization: oviposition behavior toward the major aliphatic acids of its host plant. Behav. Genet 28, 455–464. 9926614 10.1023/a:1021689312582

[19] Farine JP, Legal L, Moreteau B, and Le Quere JL (1996). Volatile components of ripe fruits of Morinda citrifolia and their effects on Drosophila. Phytochemistry 41, 433–438. 10.1016/0031-9422(95)00455-6.

[20] Jones CD (1998). The genetic basis of Drosophila sechellia’s resistance to a host plant toxin. Genetics 149, 1899–1908. 10.1093/genetics/149.4.1899. 9691045 PMC1460277

[21] Lanno SM, Gregory SM, Shimshak SJ, Alverson MK, Chiu K, Feil AL, Findley MG, Forman TE, Gordon JT, Ho J, (2017). Transcriptomic analysis of octanoic acid response in Drosophila sechellia using RNA-sequencing. G3 (Bethesda) 7, 3867–3873. 10.1534/g3.117.300297. 29021218 PMC5714484

[22] Lanno SM, Lam I, Drum Z, Linde SC, Gregory SM, Shimshak SJ, Becker MV, Brew KE, Budhiraja A, Carter EA, (2019). Genomics analysis of L-DOPA exposure in Drosophila sechellia. G3 (Bethesda) 9, 3973–3980. 10.1534/g3.119.400552. 31575638 PMC6893205

[23] Andrade López JM, Lanno SM, Auerbach JM, Moskowitz EC, Sligar LA, Wittkopp PJ, and Coolon JD (2017). Genetic basis of octanoic acid resistance in Drosophila sechellia: functional analysis of a fine-mapped region. Mol. Ecol 26, 1148–1160. 10.1111/mec.14001. 28035709 PMC5330365

[24] Jones CD (2001). The genetic basis of larval resistance to a host plant toxin in Drosophila sechellia. Genet. Res 78, 225–233. 10.1017/s0016672301005298. 11865712

[25] Hungate EA, Earley EJ, Boussy IA, Turissini DA, Ting CT, Moran JR, Wu ML, Wu CI, and Jones CD (2013). A locus in Drosophila sechellia affecting tolerance of a host plant toxin. Genetics 195, 1063–1075. 10.1534/genetics.113.154773. 24037270 PMC3813837

[26] Shah N, Dorer DR, Moriyama EN, and Christensen AC (2012). Evolution of a large, conserved, and syntenic gene family in insects. G3 (Bethesda) 2, 313–319. 10.1534/g3.111.001412. 22384409 PMC3284338

[27] Scholl A, Ndoja I, Dhakal N, Morante D, Ivan A, Newman D, Mossington T, Clemans C, Surapaneni S, Powers M, and Jiang L (2023). The Osiris family genes function as novel regulators of the tube maturation process in the Drosophila trachea. PLoS Genet 19, e1010571. 10.1371/journal.pgen.1010571. 36689473 PMC9870157

[28] Lee J, Song M, and Hong S (2013). Negative regulation of the novel norpA(P24) suppressor, diehard4, in the endo-lysosomal trafficking underlies photoreceptor cell degeneration. PLoS Genet 9, e1003559. 10.1371/journal.pgen.1003559. 23754968 PMC3674991

[29] Ando T, Sekine S, Inagaki S, Misaki K, Badel L, Moriya H, Sami MM, Itakura Y, Chihara T, Kazama H, (2019). Nanopore formation in the cuticle of an insect olfactory sensillum. Curr. Biol 29, 1512–1520.e6. 10.1016/j.cub.2019.03.043. 31006566

[30] Scalzotto M, Ng R, Cruchet S, Saina M, Armida J, Su CY, and Benton R (2022). Pheromone sensing in Drosophila requires support cell-expressed Osiris 8. BMC Biol 20, 230. 10.1186/s12915-022-01425-w. 36217142 PMC9552441

[31] Lanno SM, Shimshak SJ, Peyser RD, Linde SC, and Coolon JD (2019). Investigating the role of Osiris genes in Drosophila sechellia larval resistance to a host plant toxin. Ecol. Evol 9, 1922–1933. 10.1002/ece3.4885. 30847082 PMC6392368

[32] Dekker T, Ibba I, Siju KP, Stensmyr MC, and Hansson BS (2006). Olfactory shifts parallel superspecialism for toxic fruit in Drosophila melanogaster sibling, D. sechellia. Curr. Biol 16, 101–109. 10.1016/j.cub.2005.11.075. 16401429

[33] Matsuo T, Sugaya S, Yasukawa J, Aigaki T, and Fuyama Y (2007). Odorant-binding proteins OBP57d and OBP57e affect taste perception and host-plant preference in Drosophila sechellia. PLoS Biol 5, e118–e206. 10.1371/journal.pbio.0050118. 17456006 PMC1854911

[34] Harada E, Haba D, Aigaki T, and Matsuo T (2008). Behavioral analyses of mutants for two odorant-binding protein genes, Obp57d and Obp57e, in Drosophila melanogaster. Genes Genet. Syst 83, 257–264. 10.1266/ggs.83.257. 18670137

[35] Dworkin I, and Jones CD (2009). Genetic changes accompanying the evolution of host specialization in Drosophila sechellia. Genetics 181, 721–736. 10.1534/genetics.108.093419. 19033155 PMC2644960

[36] Matsuo T (2012). Contribution of olfactory and gustatory sensations of octanoic acid in the oviposition behavior of Drosophila melanogaster (Diptera: Drosophilidae). Appl. Entomol. Zool 47, 137–142.

[37] Auer TO, Khallaf MA, Silbering AF, Zappia G, Ellis K, Álvarez-Ocaña R, Arguello JR, Hansson BS, Jefferis GSXE, Caron SJC, (2020). Olfactory receptor and circuit evolution promote host specialization. Nature 579, 402–408. 10.1038/s41586-020-2073-7. 32132713 PMC7100913

[38] Prieto-Godino LL, Rytz R, Cruchet S, Bargeton B, Abuin L, Silbering AF, Ruta V, Dal Peraro M, and Benton R (2017). Evolution of acid-sensing olfactory circuits in drosophilids. Neuron 93, 661–676.e6. 10.1016/j.neuron.2016.12.024. 28111079

[39] Masek P, and Keene AC (2013). Drosophila fatty acidtaste signals through the PLC pathway in sugar-sensing neurons. PLoS Genet 9, e1003710, [pii]. 10.1371/journal.pgen.1003710PGENETICS-D-13-00713. 24068941 PMC3772025

[40] Tauber JM, Brown EB, Li Y, Yurgel ME, Masek P, and Keene AC (2017). A subset of sweet-sensing neurons identified by IR56d are necessary and sufficient for fatty acid taste. PLoS Genet 13, e1007059. 10.1371/journal.pgen.1007059. 29121639 PMC5697886

[41] Ahn JE, Chen Y, and Amrein H (2017). Molecular basis of fatty acid taste in Drosophila. Elife 6, e30115. 10.7554/eLife.30115. 29231818 PMC5747521

[42] Sánchez-Alcañiz JA, Silbering AF, Croset V, Zappia G, Sivasubramaniam AK, Abuin L, Sahai SY, Münch D, Steck K, Auer TO, (2018). An expression atlas of variant ionotropic glutamate receptors identifies a molecular basis of carbonation sensing. Nat. Commun 9, 4252. 10.1038/s41467-018-06453-1. 30315166 PMC6185939

[43] Higa I, and Fuyama Y (1993). Genetics of food preference in Drosophila sechellia. I. Responses to food attractants. Genetica 88, 129–136. 10.1007/BF02424469. 8224853

[44] Swarup S, Morozova TV, Sridhar S, Nokes M, and Anholt RRH (2014). Modulation of feeding behavior by odorant-binding proteins in Drosophila melanogaster. Chem. Senses 39, 125–132. 10.1093/chemse/bjt061. 24302688 PMC3894858

[45] Galindo K, and Smith DP (2001). A large family of divergent Drosophila odorant-binding proteins expressed in gustatory and olfactory sensilla. Genetics 159, 1059–1072. 10.1093/genetics/159.3.1059. 11729153 PMC1461854

[46] Jeong YT, Shim J, Oh SR, Yoon HI, Kim CH, Moon SJ, and Montell C (2013). An odorant-binding protein required for suppression of sweet taste by bitter chemicals. Neuron 79, 725–737, [pii]. 10.1016/j.neuron.2013.06.025S0896-6273(13)00541-2. 23972598 PMC3753695

[47] Rihani K, Fraichard S, Chauvel I, Poirier N, Delompré T, Neiers F, Tanimura T, Ferveur JF, and Briand L (2019). A conserved odorant binding protein is required for essential amino acid detection in Drosophila. Commun. Biol 2, 425. 10.1038/s42003-019-0673-2. 31799428 PMC6874667

[48] Matsuo T (2008). Genes for host-plant selection in Drosophila. J. Neurogenet 22, 195–210. 10.1080/01677060802298483. 19040187

[49] Kim H, Kim H, Kwon JY, Seo JT, Shin DM, and Moon SJ (2018). Drosophila Gr64e mediates fatty acid sensing via the phospholipase C pathway. PLoS Genet 14, e1007229. 10.1371/journal.pgen.1007229. 29420533 PMC5821400

[50] Brown EB, Shah KD, Palermo J, Dey M, Dahanukar A, and Keene AC (2021). Ir56d-dependent fatty acid responses in Drosophila uncover taste discrimination between different classes of fatty acids. Elife 10, e67878. 10.7554/eLife.67878. 33949306 PMC8169106

[51] Larsson MC, Domingos AI, Jones WD, Chiappe ME, Amrein H, and Vosshall LB (2004). Or83b encodes a broadly expressed odorant receptor essential for Drosophila olfaction. Neuron 43, 703–714. 15339651 10.1016/j.neuron.2004.08.019

[52] Dweck HKM, and Carlson JR (2020). Molecular logic and evolution of bitter taste in Drosophila. Curr. Biol 30, 17–30.e3. 10.1016/j.cub.2019.11.005. 31839451 PMC6946858

[53] Weiss LA, Dahanukar A, Kwon JY, Banerjee D, and Carlson JR (2011). The molecular and cellular basis of bitter taste in Drosophila. Neuron 69, 258–272, S0896-6273(11)00002-X [pii]. 10.1016/j.neuron.2011.01.001. 21262465 PMC3033050

[54] Devineni AV, Sun B, Zhukovskaya A, and Axel R (2019). Acetic acid activates distinct taste pathways in Drosophila to elicit opposing, state-dependent feeding responses. Elife 8, e47677. 10.7554/eLife.47677. 31205005 PMC6579511

[55] Stanley M, Ghosh B, Weiss ZF, Christiaanse J, and Gordon MD (2021). Mechanisms of lactic acid gustatory attraction in Drosophila. Curr. Biol 31, 3525–3537.e6. 10.1016/j.cub.2021.06.005. 34197729

[56] Pradhan RN, Shrestha B, and Lee Y (2023). Molecular basis of hexanoic acid taste in Drosophila melanogaster. Mol. Cells 46, 451–460. 10.14348/molcells.2023.0035. 37202372 PMC10336273

[57] Charlu S, Wisotsky Z, Medina A, and Dahanukar A (2013). Acid sensing by sweet and bitter taste neurons in Drosophila melanogaster. Nat. Commun 4, 2042, [pii]. 10.1038/ncomms3042ncomms3042. 23783889 PMC3710667

[58] Chen YCD, Park SJ, Joseph RM, Ja WW, and Dahanukar AA (2019). Combinatorial pharyngeal taste coding for feeding avoidance in adult Drosophila. Cell Rep 29, 961–973.e4. 10.1016/j.cel-rep.2019.09.036. 31644916 PMC6860367

[59] French AS, Sellier MJ, Ali Agha M, Guigue A, Chabaud MA, Reeb PD, Mitra A, Grau Y, Soustelle L, and Marion-Poll F (2015). Dual mechanism for bitter avoidance in Drosophila. J. Neurosci 35, 3990–4004, [pii]. 10.1523/JNEUROSCI.1312-14.201535/9/3990. 25740527 PMC6605581

[60] Sellier MJ, Reeb P, and Marion-Poll F (2011). Consumption of bitter alkaloids in Drosophila melanogaster in multiple-choice test conditions. Chem. Senses 36, 323–334. 10.1093/chemse/bjq133. 21173029

[61] Meunier N, Marion-Poll F, Rospars JP, and Tanimura T (2003). Peripheral coding of bitter taste in Drosophila. J. Neurobiol 56, 139–152. 12838579 10.1002/neu.10235

[62] Chen HL, Stern U, and Yang CH (2019). Molecular control limiting sensitivity of sweet taste neurons in Drosophila. Proc. Natl. Acad. Sci. USA 116, 20158–20168. 10.1073/pnas.1911583116. 31527261 PMC6778258

[63] Koh TW, He Z, Gorur-Shandilya S, Menuz K, Larter NK, Stewart S, and Carlson JR (2014). The Drosophila IR20a clade of ionotropic receptors are candidate taste and pheromone receptors. Neuron 83, 850–865, [pii]. 10.1016/j.neuron.2014.07.012S0896-6273(14)00623-0. 25123314 PMC4141888

[64] Chen YCD, and Dahanukar A (2017). Molecular and cellular organization of taste neurons in adult Drosophila pharynx. Cell Rep 21, 2978–2991. 10.1016/j.celrep.2017.11.041. 29212040 PMC5736000

[65] van Giesen L, and Garrity PA (2017). More than meets the IR: the expanding roles of variant Ionotropic Glutamate Receptors in sensing odor, taste, temperature and moisture. F1000Res 6, 1753. 10.12688/f1000research.12013.1. 29034089 PMC5615767

[66] Croset V, Rytz R, Cummins SF, Budd A, Brawand D, Kaessmann H, Gibson TJ, and Benton R (2010). Ancient protostome origin of chemosensory ionotropic glutamate receptors and the evolution of insect taste and olfaction. PLoS Genet 6, e1001064. 10.1371/journal.pgen.1001064. 20808886 PMC2924276

[67] Yassin A, Debat V, Bastide H, Gidaszewski N, David JR, and Pool JE (2016). Recurrent specialization on a toxic fruit in an island Drosophila population. Proc. Natl. Acad. Sci. USA 113, 4771–4776. 10.1073/pnas.1522559113. 27044093 PMC4855561

[68] Yassin A (2017). Drosophila yakuba mayottensis, a new model for the study of incipient ecological speciation. Fly (Austin) 11, 37–45. 10.1080/19336934.2016.1221550. 27560369 PMC6516792

[69] McBride CS (2007). Rapid evolution of smell and taste receptor genes during host specialization in Drosophila sechellia. Proc. Natl. Acad. Sci. USA 104, 4996–5001. 10.1073/pnas.0608424104. 17360391 PMC1829253

[70] McBride CS, Arguello JR, and O’Meara BC (2007). Five Drosophila genomes reveal nonneutral evolution and the signature of host specialization in the chemoreceptor superfamily. Genetics 177, 1395–1416. 10.1534/genetics.107.078683. 18039874 PMC2147975

[71] Wisotsky Z, Medina A, Freeman E, and Dahanukar A (2011). Evolutionary differences in food preference rely on Gr64e, a receptor for glycerol. Nat. Neurosci 14, 1534–1541, nn.2944 [pii]. 10.1038/nn.2944. 22057190

[72] Prieto-Godino LL, Rytz R, Bargeton B, Abuin L, Arguello JR, Peraro MD, and Benton R (2016). Olfactory receptor pseudo-pseudogenes. Nature 539, 93–97. 10.1038/nature19824. 27776356 PMC5164928

[73] Álvarez-Ocaña R, Shahandeh MP, Ray V, Auer TO, Gompel N, and Benton R (2023). Odor-regulated oviposition behavior in an ecological specialist. Nat. Commun 14, 3041. 10.1038/s41467-023-38722-z. 37236992 PMC10219952

[74] Bontonou G, Saint-Leandre B, Kafle T, Baticle T, Hassan A, Sanchez-Alcaniz JA, and Arguello JR (2023). Evolution of chemosensory tissues and cells across ecologically diverse drosophilids. Preprint at bioRxiv, 2023.2004. 2014.536691. 10.1101/2023.04.14.536691. PMC10844241 38316749

[75] Vieira FG, Sánchez-Gracia A, and Rozas J (2007). Comparative genomic analysis of the odorant-binding protein family in 12 Drosophila genomes: purifying selection and birth-and-death evolution. Genome Biol 8, R235. 10.1186/gb-2007-8-11-r235. 18039354 PMC2258175

[76] Ferreira EA, Lambert S, Verrier T, Marion-Poll F, and Yassin A (2020). Soft selective sweep on chemosensory genes correlates with ancestral preference for toxic noni in a specialist Drosophila population. Genes 12, 32. 10.3390/genes12010032. 33383708 PMC7824377

[77] Reisenman CE, Wong J, Vedagarbha N, Livelo C, and Scott K (2023). Taste adaptations associated with host specialization in the specialist Drosophila sechellia. J. Exp. Biol 226, jeb244641. 10.1242/jeb.244641. 36637369 PMC10088416

[78] Dweck HKM, Talross GJS, Luo Y, Ebrahim SAM, and Carlson JR (2022). Ir56b is an atypical ionotropic receptor that underlies appetitive salt response in Drosophila. Curr. Biol 32, 1776–1787.e4. 10.1016/j.cub.2022.02.063. 35294865 PMC9050924

[79] Dahanukar A, and Benton R (2023). Recording from fly taste sensilla. Cold Spring Harb. Protoc 2023, pdb.prot108064. 10.1101/pdb.prot108064. 36446534

