# Supplementary material for: Evolution of fatty acid taste in drosophilids
Source: Cell Rep. Author manuscript; Available in PMC 2023 Dec 5. (PMC10697176; doi:10.1016/j.celrep.2023.113297)
Supplement: 1 [file NIHMS1941907-supplement-1.pdf]

Cell Reports, Volume *42*

Supplemental information

Evolution of fatty acid taste in drosophilids

Manali Dey, Elizabeth Brown, Sandhya Charlu, Alex Keene, and Anupama Dahanukar

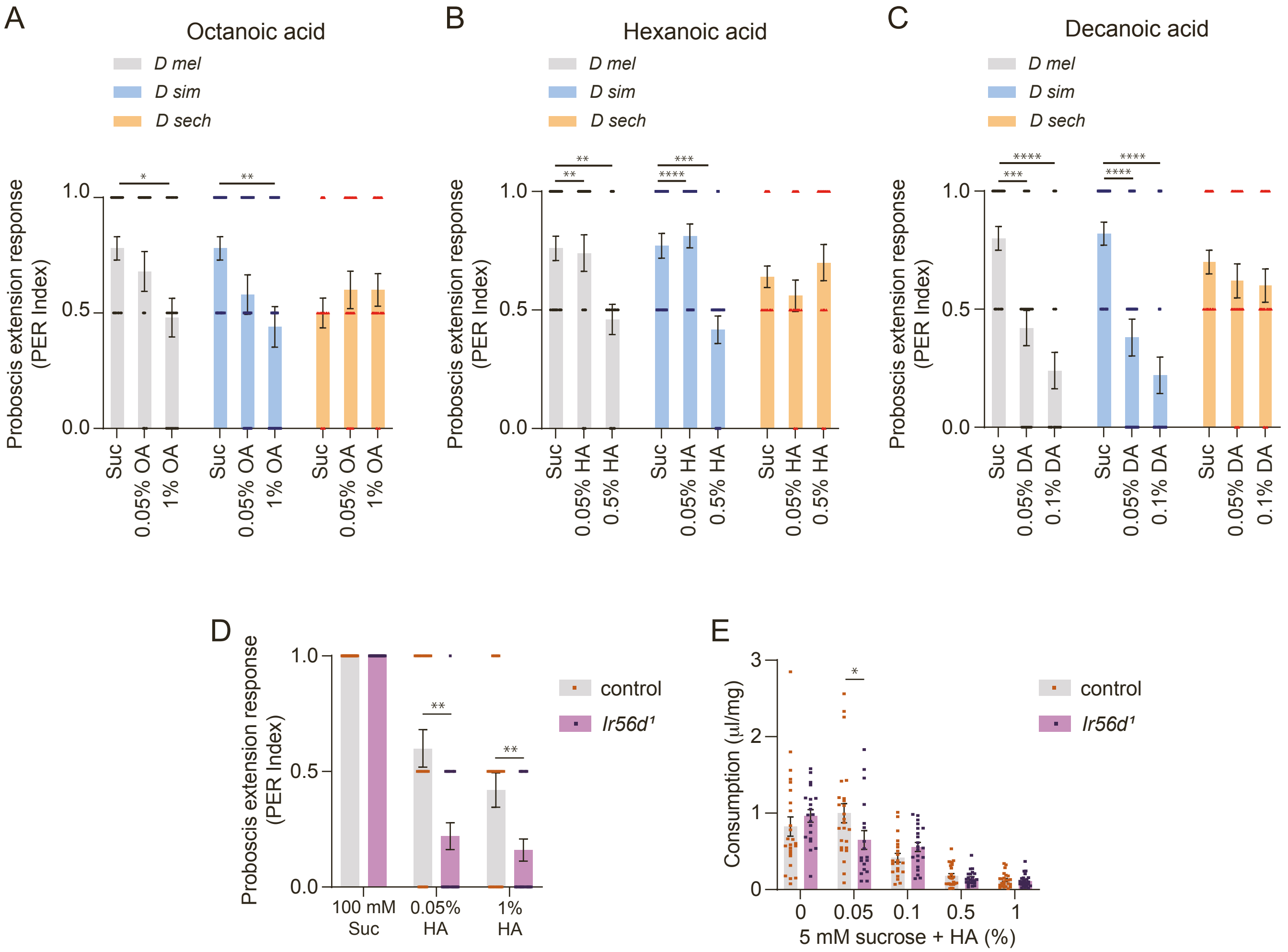

Figure S1, Related to Figure 1. Noni FAs elicit proboscis extension in generalist and specialist species.

A) Proboscis extension response of three species to sucrose (30 mM) or octanoic acid (OA) at two different concentrations. *n*=24. **B)** Proboscis extension response of three species to sucrose (30 mM) or hexanoic acid (HA) at two different concentrations. *n*=25. **C)** Proboscis extension response of three species to sucrose (30 mM) or decanoic acid (DA) at two different concentrations. *n*=25. **D)** Proboscis extension responses to indicated concentrations of sucrose (suc) or hexanoic acid (HA). *n*=25. **E)** Ingested volume (µl) of indicated diet normalized to body weight (mg) in mated females. *n*=18-25. Genotypes in D and E were: *Ir56d* parental line (control), and *Ir56d*^{1}. Data in A-D were analyzed using Friedman's test; data in E were analyzed using two-way ANOVA with Tukey's *post hoc* multiple comparisons test. *p<0.05, **p<0.01, ***p<0.001, ****p<0.0001. Error bars represent SEM.

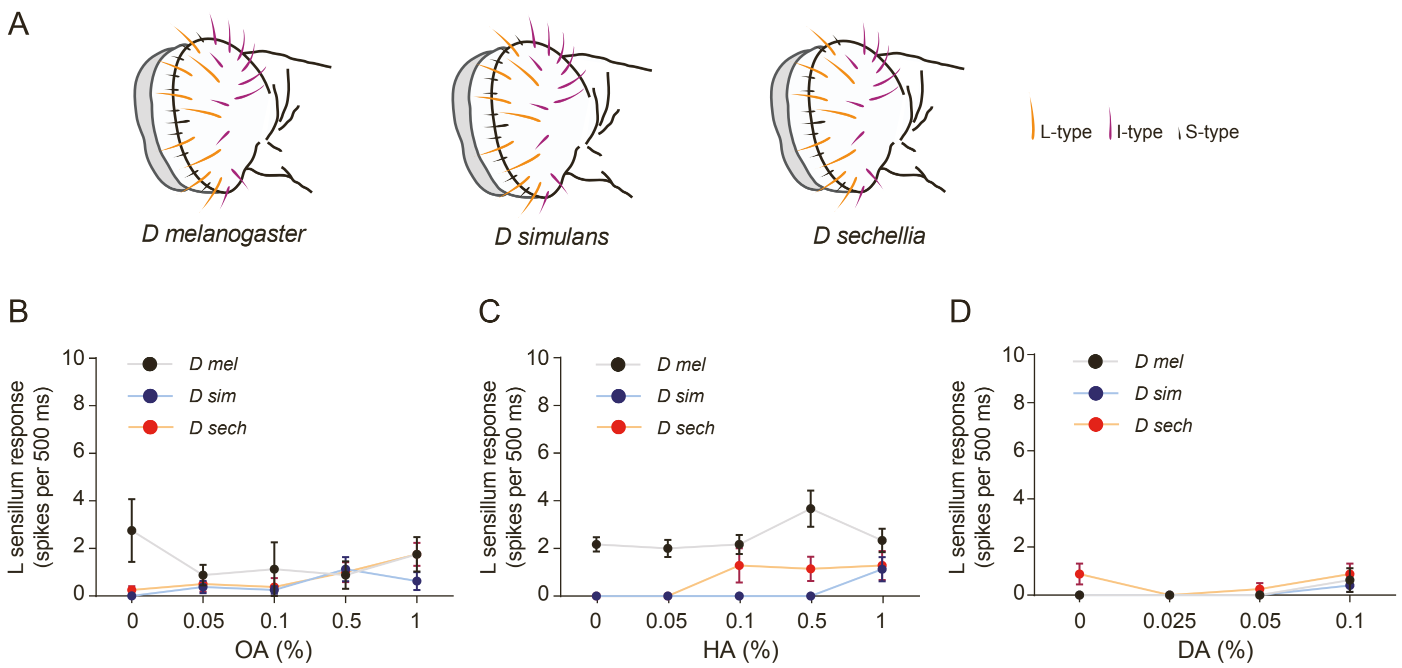

Figure S2, Related to Figure 3. Noni FAs do not elicit robust activation of selected L-type sensilla.

A) Arrangement of sensillar types in the labella of three *Drosophila* species. **B-D)** Responses of labellar L-type sensilla in indicated species to varying concentrations of octanoic acid (OA; B, *n*=8), hexanoic acid (HA; C, *n*=6-8), and decanoic acid (DA; D, *n*=8).

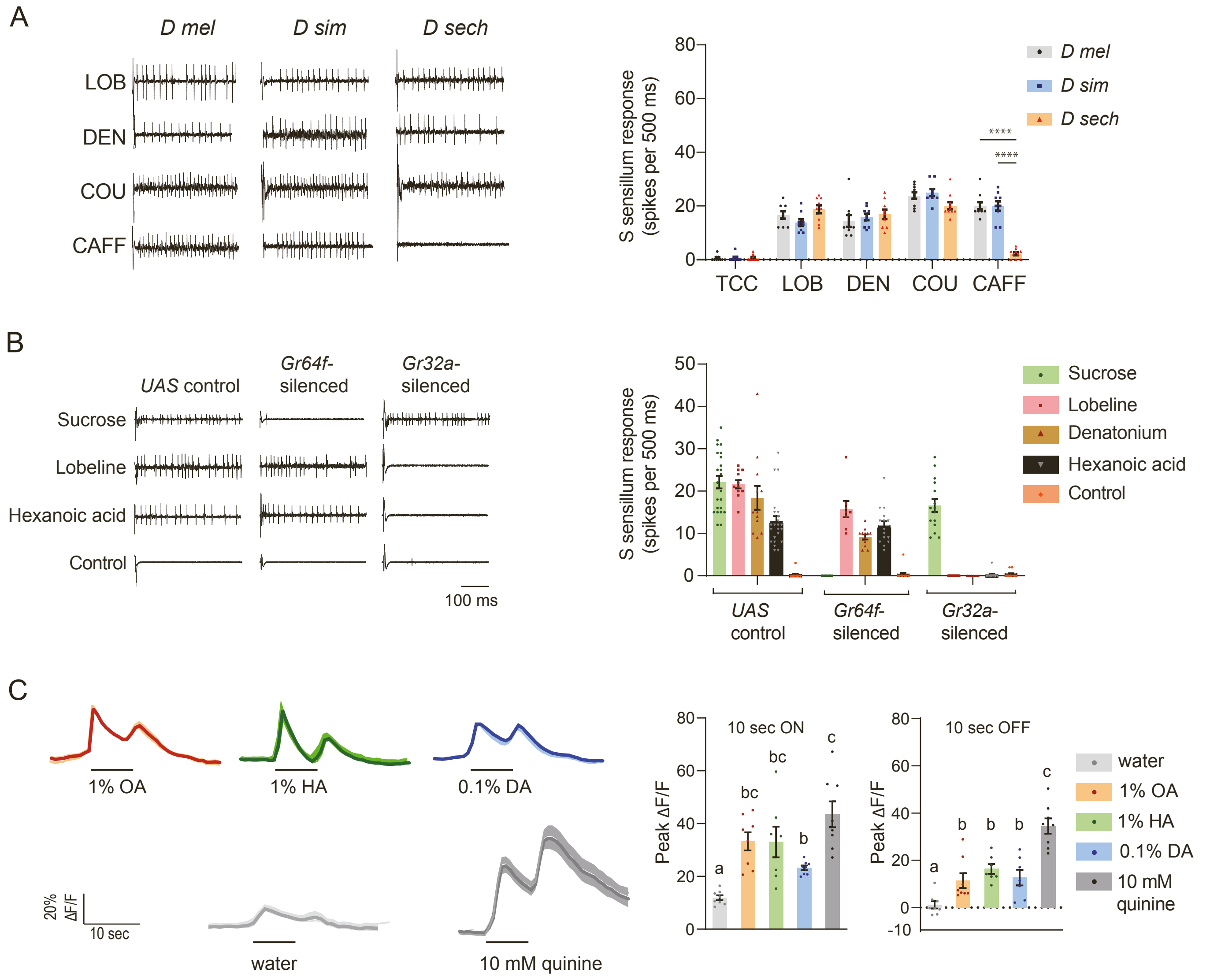

Figure S3, Related to Figure 3. Bitter neurons in S-type sensilla are activated by noni FAs.

A) Representative traces (left) and mean responses (right) of labellar S-type sensilla in indicated species upon stimulation with 10 mM of bitter tastants: lobeline (LOB), denatonium (DEN), coumarin (COU) and caffeine (CAFF). *n*=9-10. **B)** Representative traces (left) and mean responses (right) obtained from S-type stimulated with the indicated tastants. Genotypes were: *UAS-Kir2.1* (*UAS* control); *Gr64f-GAL4>UAS-Kir2.1* (*Gr64f*-silenced); *Gr32a-GAL4>UAS-Kir2.1* (*Gr32a*-silenced). Stimuli were: sucrose (100 mM), lobeline (10 mM), denatonium (10 mM), hexanoic acid (1%), and tricholine citrate diluent control (30 mM). For *UAS* control flies, *n*=23 (sucrose), *n*=11 (lobeline), *n*=12 (denatonium), *n*=23 (hexanoic acid), *n*=23 (control). For *Gr64f*-silenced flies, *n*=23 (sucrose), *n*=11 (lobeline), *n*=12 (denatonium), *n*=23 (hexanoic acid), *n*=15 (control). For *Gr32a*-silenced flies, *n*=15 (sucrose), *n*=15 (lobeline), *n*=6 (denatonium), *n*=15 (hexanoic acid), *n*=15 (control). For each stimulus*genotype, recordings were taken from 5-7 flies. **C)** Activity traces and average peak changes in GCaMP6 fluorescence in *Gr66a+* neurons during (10 sec ON) and immediately after (10 sec OFF) 10-sec applications of indicated tastants. *n*=7-8. For activity traces, the shaded region indicates ±s.e.m. and period of stimulus application is indicated by the black bar below the trace. Data in A and B were analyzed using two-way ANOVA with Tukey's *post hoc* multiple comparisons test. Data in C were analyzed with 1-way ANOVA with Sidak's *post hoc* test for multiple comparisons. For all graphs, *$p<0.05$, **$p<0.01$, ***$p<0.001$, ****$p<0.0001$. Error bars represent SEM.

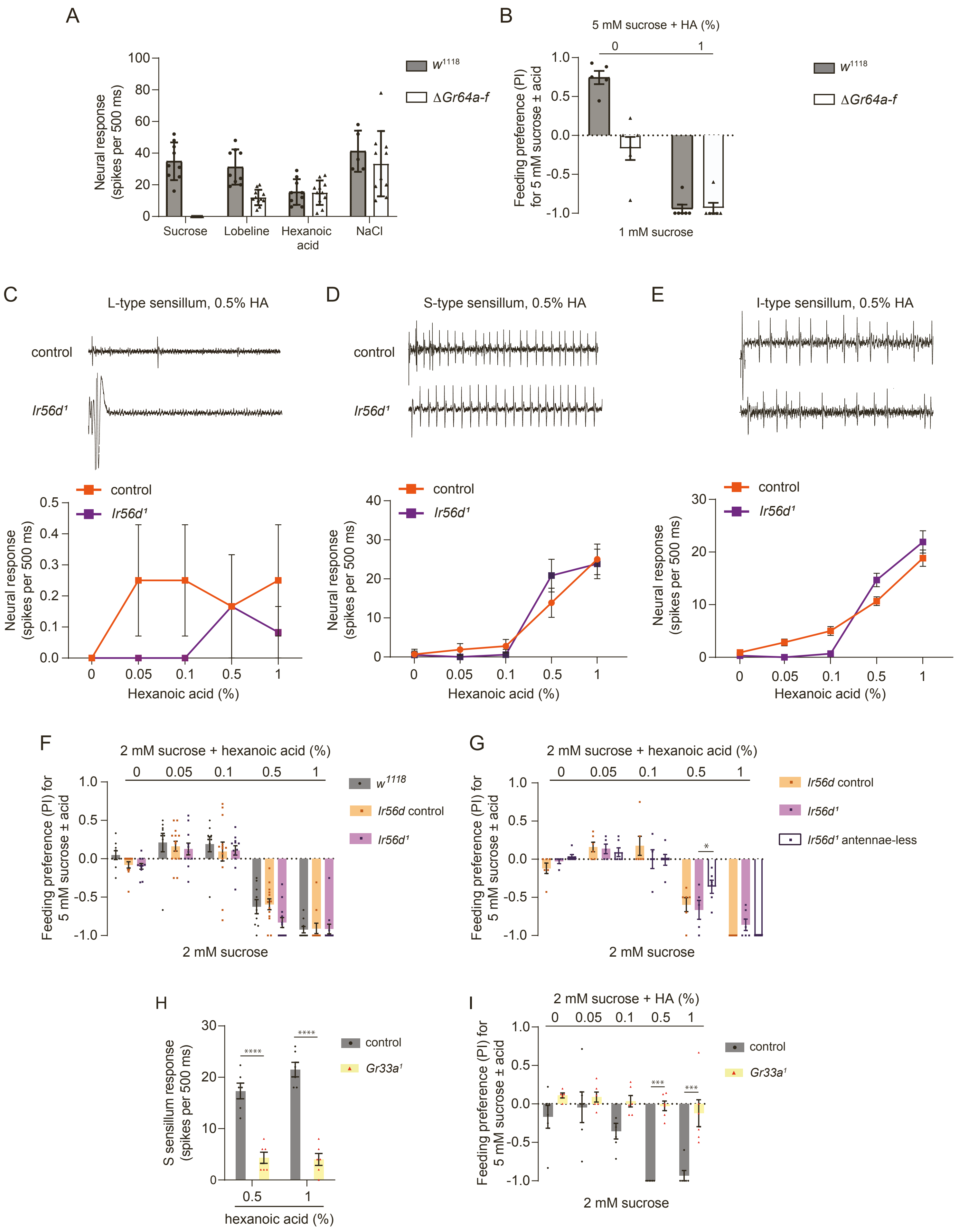

A
Neural response (spikes per 500 ms)
Sucrose
Lobeline
Hexanoic acid
NaCl
w1118
ΔGr64a-f
B
5 mM sucrose + HA (%)
Feeding preference (PI) for 5 mM sucrose ± acid
1 mM sucrose
C
L-type sensillum, 0.5% HA
control
Ir56d1
D
S-type sensillum, 0.5% HA
E
I-type sensillum, 0.5% HA
Neural response (spikes per 500 ms)
Hexanoic acid (%)
F
2 mM sucrose + hexanoic acid (%)
Ir56d control
2 mM sucrose
G
Ir56d1 antennae-less
H
S sensillum response (spikes per 500 ms)
hexanoic acid (%)
Gr33a1
I
2 mM sucrose + HA (%)

Figure S4, Related to Figure 3. HA-evoked neuronal responses and behavioral aversion are retained in *Gr64a-f* and *Ir56d* mutants but not in *Gr33a* mutants.
A) Mean responses obtained from labellar S-type sensilla stimulated with the indicated tastants. Genotypes were: w^{1118} and Δ*Gr64a-f*. Stimuli were sucrose (100 mM), lobeline (10 mM), hexanoic acid (1%), and NaCl (500 mM). *n*=9-13. B) Feeding preference of w^{1118} and Δ *Gr64a-f* flies for 5 mM sucrose mixed with hexanoic acid (HA) tested against 1 mM sucrose. *n*=5-6. **C-E)** Representative traces (top) and mean responses (bottom) of L-type (C, n=12), S-type (D, n=9-12) and I-type (E, n=13) sensilla to indicated concentrations of hexanoic acid (HA). **F)** Feeding preference for mixtures of 2 mM sucrose with varying concentrations of hexanoic acid tested against 2 mM sucrose. *n*=9-12. **G)** Feeding preference for mixtures of 2 mM sucrose with varying concentrations of hexanoic acid tested against 2 mM sucrose. *n*=6. Genotypes in C-G were: w^{1118}, *Ir56d* parental line (*Ir56d* control), and $Ir56d^{1}$. **H)** Responses of labellar S-type sensilla of w^{1118} and $Gr33a^{1}$ flies stimulated with indicated concentrations of hexanoic acid (HA). *n*=6. **I)** Feeding preference for mixtures of 2 mM sucrose with varying concentrations of hexanoic acid (HA) tested against 2 mM sucrose. Genotypes as in H. *n*=6. Data in A-E and H-I were analyzed using two-way ANOVA with Sidak's *post hoc* multiple comparisons test; data in F and G were analyzed using two-way ANOVA for repeated measures followed by Tukey's *post hoc* test for multiple comparisons. *$p<0.05$, **$p<0.01$, ***$p<0.001$, ****$p<0.0001$. Error bars represent SEM.

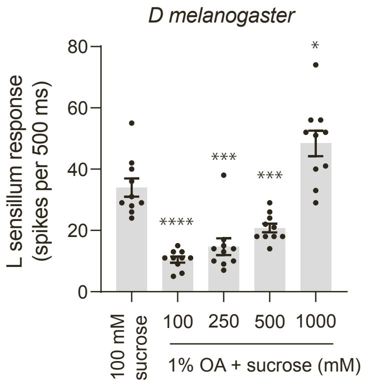

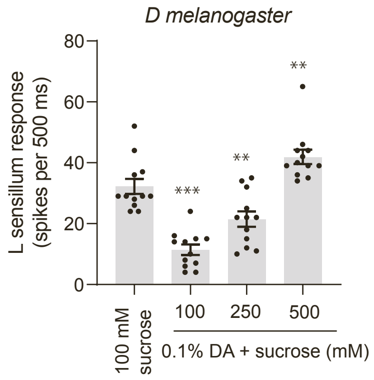

Figure S5, Related to Figure 4. Noni FA-mediated inhibition of sweet taste is overcome by increasing sugar concentration.

Mean responses of labellar L-type sensilla in *D. melanogaster* stimulated with sucrose alone (100 mM) or indicated concentrations mixed with 1% OA (top) or 0.1% DA (bottom). n=10-12. Data were analyzed using Mann Whitney test, *p<0.05, **p<0.01, ***p<0.001, ****p<0.0001. Error bars represent SEM.
